# A Primate‐Specific lncRNA *LINC01021* Contributes to Cellular and Organismal Aging via DAZAP1‐Dependent Destabilization of RBMX

**DOI:** 10.1111/acel.70603

**Published:** 2026-06-25

**Authors:** Yan Zhang, Li Hu, Xin Dong, Qinghua Zeng, Meiting Zi, Ayesha Nisar, Sawar Khan, Raoxian Bai, Chonghui Liu, Mingxia Ge, Shaoyan Pu, Gonghua Li, Yonghan He

**Affiliations:** ^1^ State Key Laboratory of Genetic Evolution & Animal Models, Key Laboratory of Healthy Aging Research of Yunnan Province Kunming Institute of Zoology, Chinese Academy of Sciences Kunming China; ^2^ University of Chinese Academy of Sciences Beijing China; ^3^ Institute of Molecular Biology and Biotechnology The University of Lahore Lahore Pakistan; ^4^ Biodiversity Data Center of Kunming Institute of Zoology Chinese Academy of Sciences Kunming China; ^5^ State Key Laboratory of Primate Biomedical Research, Institute of Primate Translational Medicine Kunming University of Science and Technology Kunming China; ^6^ School of Basic Medical Sciences Wannan Medical College Wuhu China; ^7^ KIZ/CUHK Joint Laboratory of Bioresources and Molecular Research in Common Diseases Kunming China

**Keywords:** aging, cellular senescence, LINC01021, lncRNA, primate‐specific lncRNAs, RBMX

## Abstract

Aging is characterized by progressive physiological decline and age‐related pathologies, yet the molecular determinants underlying lineage‐ and species‐specific aging traits remain poorly understood. Although protein‐coding regulators have dominated aging research, the contribution of long non‐coding RNAs (lncRNAs), particularly primate‐specific lncRNAs, has not been systematically explored. Here, through evolutionary screening and cross‐species aging‐associated analyses, we identified a set of primate‐specific lncRNAs (including *LINC01021*, *CTC‐575 l10.1*, *CTA‐150C2.13*, and *RP11‐305F18.1*, etc.) associated with human aging, and we functionally characterized *LINC01021* as a representative candidate to assess their causal involvement. In human cells, *LINC01021* promotes cellular senescence, whereas its silencing attenuates senescence‐associated phenotypes. Mechanistically, *LINC01021* is predominantly located in the nucleus, where it facilitates DAZAP1‐dependent destabilization of RBMX mRNA, leading to activation of the P53 pathway and induction of canonical senescence features. At the organismal level, ectopic expression of human *LINC01021* in mice contributes to aging‐like phenotypes, including increased frailty and impaired motor coordination. Together, these findings implicate primate‐specific lncRNAs in lineage‐restricted aging and highlight an evolutionarily recent regulatory layer that may modulate aging trajectories.

## Graphical abstract

Primate‐specific lncRNA *LINC01021* promotes aging by enhancing cellular senescence and accelerating aspects of organismal decline. Mechanistically, *LINC01021* induces DAZAP1‐mediated destabilization of RBMX, activating the P53 pathway and establishing a primate‐specific regulatory axis in aging.

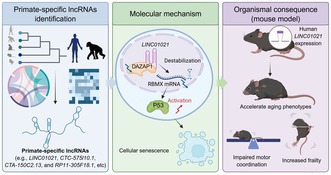

**Abbreviations**
Act D: Actinomycin D
ANOVA: Analysis of variance
BJ: BJ foreskin fibroblast
ChIP: Chromatin immunoprecipitation
CHX: Cycloheximide
DOX: Doxorubicin
FC: Fold change
FI: Frailty index
GAM: Generalized additive model
GEO: Gene Expression Omnibus
gRNA: Guide RNA
GTEx: Genotype‐tissue expression
HDF: Human dermal fibroblast
HELF: Human embryonic lung fibroblast
HFF: Human foreskin fibroblast
IMR90: IMR‐90 fetal lung fibroblast
IR: Ionizing radiation
KI: Knock‐in
*LINC01021*‐OE: *LINC01021* overexpression
lncRNA: Long non‐coding RNA
MRC5: MRC‐5 lung fibroblast
MS: Mass spectrometry
NcRNA: Non‐coding RNA
NIR: Near‐ infrared
qPCR: Quantitative PCR
qRT‐PCR: Quantitative real‐time PCR
RBC: Red blood cell
RBH: Reciprocal best hit
RNA‐seq: RNA sequencing
ROI: Region of interest
SASP: Senescence‐associated secretory phenotype
SA‐β‐Gal: Senescence‐associated β‐galactosidase
SCF: SKP1‐CUL1‐F‐box
SEM: Standard error of the mean
SnC: Senescent cell
SPF: Specific pathogen‐free
WBC: White blood cell

## 1 Introduction

Aging is a complex, multifaceted biological process marked by progressive physiological decline and increased vulnerability to chronic diseases (Pal and Tyler 2016). It is driven by an interconnected set of hallmarks, including genomic instability, telomere attrition, epigenetic alterations, loss of proteostasis, deregulated nutrient sensing, mitochondrial dysfunction, cellular senescence, stem‐cell exhaustion, and disrupted intercellular communication, which together shape tissue dysfunction and age‐related pathologies (López‐Otín et al. 2023). These hallmarks reflect the cumulative outcome of dynamic regulatory processes that operate across multiple molecular layers (López‐Otín et al. 2023). Although aging research has traditionally emphasized protein‐coding genes and canonical signaling pathways, growing evidence indicates that additional, under‐characterized regulatory layers make important contributions to aging biology (X. Li et al. 2023; López‐Otín et al. 2023; Wagner et al. 2024).

Non‐coding RNAs (ncRNAs) have emerged as a prominent, yet incompletely explored, regulatory layer in aging (Guo et al. 2022; Wagner et al. 2024; K. Wang et al. 2022). NcRNAs are broadly classified into small ncRNAs and long ncRNAs (lncRNAs) (Leng et al. 2024; Mattick et al. 2023; Statello et al. 2021). Small ncRNAs such as microRNAs primarily act post‐transcriptionally to modulate mRNA stability and translation, thereby fine‐tuning pathways involved in cell‐cycle control, stress responses, and metabolic homeostasis (Turko et al. 2025). By contrast, lncRNAs, transcripts longer than 200 nucleotides with limited protein‐coding potential, exhibit remarkable structural and functional versatility (Kopp and Mendell 2018; Yao et al. 2019). LncRNAs regulate gene expression at multiple levels: they recruit chromatin modifiers to shape epigenetic states, scaffold transcriptional regulators to control transcription, and influence post‐transcriptional events through interactions with microRNAs and RNA‐binding proteins; in some cases, they even encode functional micropeptides (Mattick et al. 2023; Yao et al. 2019). Because of this multilayered regulatory potential, lncRNAs are well positioned to coordinate large gene networks and to integrate diverse aging‐relevant processes such as DNA repair, inflammation, and cell‐cycle regulation (López‐Otín et al. 2023; Sherazi et al. 2023; Statello et al. 2021). Consistent with a systems‐level role, lncRNAs constitute a major fraction of the ncRNA transcriptome and are frequently dysregulated with age across multiple tissues (Marttila et al. 2020; Mattick et al. 2023; Mercer et al. 2009; Sherazi et al. 2023).

Explaining how conserved molecular processes produce dramatically different aging trajectories across species remains an unresolved challenge. Maximum lifespan varies dramatically across mammals, spanning from approximately 2–4 years in mice to over 80 years in humans and more than 200 years in bowhead whales (Firsanov et al. 2025; Keane et al. 2015; López‐Otín et al. 2023; Seluanov et al. 2018), yet protein‐coding genes are highly conserved across species, suggesting that coding sequence variation alone cannot account for such divergence (Breschi et al. 2017; Yue et al. 2014). In contrast, lncRNAs display greater evolutionary plasticity, with rapid sequence turnover, frequent lineage‐restricted emergence, and flexible regulatory roles (Mattick et al. 2023; Statello et al. 2021; Yao et al. 2019). Large‐scale comparative genomic studies show that total ncRNA content, driven primarily by lncRNAs, positively correlates with maximum lifespan across mammals, whereas protein‐coding sequence length does not show a similar positive relationship (A. Wang 2025). Notably, the human genome contains an approximately 2.7‐fold expansion of lncRNA sequence compared with the mouse, which implicates lncRNA innovation as a plausible contributor to the evolution of extended lifespan and species‐specific aging phenotypes (A. Wang 2025). Comparative investigations of exceptionally long‐lived species (e.g., naked mole‐rats, bats, and bowhead whales) further suggest that lineage‐specific regulatory innovations, rather than conserved protein‐coding changes alone, may underlie species‐restricted aging trajectories, with rapidly evolving lncRNAs representing a particularly compelling candidate layer (Cagan et al. 2022; Jiang and Kong 2020; Seluanov et al. 2018; Yu et al. 2021).

Primate lineages, and humans in particular, exhibit distinctive aging features, such as pronounced cortical expansion, extended post‐reproductive lifespan, and elevated susceptibility to neurodegenerative diseases, that are thought to arise primarily from lineage‐specific regulatory architectures rather than protein‐coding differences (Field et al. 2019; Pollen et al. 2023; Rigby Dames et al. 2023; Vickery et al. 2024). Supporting this view, multiple primate‐specific or primate‐enriched lncRNAs have been linked to aging‐relevant processes. For example, the primate‐specific lncRNA *PCAT14*, derived from human‐specific HERV‐H elements, is highly expressed and nuclear‐localized in young human endothelial cells but is downregulated during endothelial aging (Drekolia et al. 2022). This change impairs migration and angiogenesis as well as increases inflammatory adhesion, all hallmarks of vascular aging that non‐primate models do not fully recapitulate (Drekolia et al. 2022). In the developing nervous system, primate‐enriched lncRNAs show tightly regulated, transient expression during human cortical differentiation and actively shape cell type‐specific programs involved in synaptogenesis and neuronal connectivity, processes that are among the earliest and most vulnerable targets of brain aging and neurodegeneration (Field et al. 2019). Likewise, primate‐cortex‐specific lncRNAs such as *LINC00507* display age‐dependent expression changes and associate with neurodegeneration‐related pathways, further linking primate regulatory innovation to both extended lifespan and increased age‐associated disease susceptibility (Mills et al. 2016; Yan et al. 2020).

Motivated by these observations, we performed an evolutionary screen for aging‐associated transcripts across species and identified a set of primate‐specific lncRNA candidates with robust age‐dependent signatures, including *LINC01021*, *CTC‐575 l10.1*, *CTA‐150C2.13*, *RP11‐305F18.1*, and *RP11‐700 N1.1*. We prioritized *LINC01021* as a top candidate for detailed functional and mechanistic dissection. Previous studies have reported that *LINC01021* can suppress P53 signaling in certain cancer contexts, primarily through MDM2‐associated mechanisms, thereby promoting cell proliferation and tumorigenesis (Kaller et al. 2024; Kaller et al. 2017; X. L. Li et al. 2017). However, our evolutionary and functional analyses reveal a striking context‐dependent duality: in aging‐associated contexts, *LINC01021* exhibits a strong positive association with P53 activation. Mechanistically, we show that *LINC01021* accelerates cellular senescence by promoting DAZAP1‐dependent destabilization of the RNA‐binding protein RBMX, thereby activating P53 signaling. Importantly, ectopic expression of human *LINC01021* in a transgenic mouse model is associated with features of accelerated functional decline, evidenced by increased frailty and impaired motor coordination.

## 2 Materials and Methods

### 2.1 LncRNA Sequence Collection

LncRNA sequences were obtained from the NONCODE database (version 6) for multiple representative species spanning primates, mammals, vertebrates, and invertebrates (Zhao et al. 2021). NONCODE annotates lncRNAs based on transcriptomic evidence, transcript structure, length, and computational assessment of coding potential (Zhao et al. 2021). *LINC01021* is annotated in NONCODE as transcript NONHSAT100800.2, comprising 1,112 nt across five exons. Additionally, the transcript exhibits a negative CNCI score (−0.1515520), supporting its low protein‐coding potential and corroborating its annotation as a lncRNA in the NONCODE database. Human tissue‐level RNA‐seq expression data were downloaded from the Genotype‐Tissue Expression (GTEx) project (Consortium 2013).

### 2.2 Pairwise Sequence Alignment and Conservation Assessment

To evaluate evolutionary conservation of lncRNAs across species, all species were subjected to exhaustive pairwise sequence comparisons. For each species pair, nucleotide‐level alignments were performed using BLASTN. For each query lncRNA, significant alignments were retained based on sequence similarity, alignment length, and coverage thresholds to ensure biologically meaningful matches. For each query transcript, only the highest‐scoring alignment in the target species was retained. Reciprocal best hit (RBH) analysis was then performed to define putative orthologous lncRNA pairs. A lncRNA pair was considered conserved between two species if each transcript represented the best hit of the other in reciprocal searches. The number of RBH‐supported lncRNA pairs was summarized for all species combinations to characterize the global conservation landscape. LncRNAs were defined as primate‐conserved if they showed reciprocal best‐hit support among primate species but lacked detectable RBH relationships in non‐primate species included in this study. These primate‐specific conserved lncRNAs were subsequently used for downstream aging‐associated expression analyses.

### 2.3 Identification of Aging‐Associated lncRNAs

Human tissue RNA‐seq expression data were obtained from the GTEx project. Genomic coordinates of primate‐conserved lncRNAs were intersected with GTEx gene annotations to identify corresponding transcripts, requiring at least 90% reciprocal overlap to ensure high‐confidence mapping. Only lncRNAs satisfying this criterion were retained for downstream analyses. For each tissue, samples with available age information were included. Age was modeled as a continuous variable, and sex was incorporated as a covariate when applicable. Lowly expressed lncRNAs were excluded before modeling to enhance statistical stability. To characterize age‐dependent expression dynamics, generalized additive models (GAMs) were fitted independently for each lncRNA within each tissue according to the following model:

$${\text{Expression}}_{i}=\beta_{0}+s\left({\mathrm{Age}}_{i}\right)+\beta_{1}{\mathrm{Sex}}_{i}+\varepsilon_{i}$$

where s(Age) represents a penalized regression spline capturing non‐linear age effects, and Sex was included when applicable. The statistical significance of the smooth age term was evaluated for each lncRNA. Multiple testing correction was performed using the Benjamini–Hochberg procedure. LncRNAs with significant age‐dependent effects after correction were defined as aging‐associated lncRNAs.

### 2.4 Cell Culture and Transfection

Human embryonic lung fibroblasts (HELFs) were obtained from the Kunming Cell Bank of Type Culture Collection (Kunming, China). Primary human dermal fibroblasts (HDFs) were isolated from circumcised foreskins of healthy male donors (aged 5–20 years), as previously described (Xiao et al. 2022). Cells were cultured in Dulbecco's modified Eagle medium (DMEM; C11995500BT, Gibco, MD, USA) supplemented with 10% fetal bovine serum (FBS; RYS‐F22‐05, Royacel Biotechnology, Lanzhou, China) and 1% penicillin–streptomycin (15140–122, Gibco, MD, USA) at 37°C in a humidified incubator with 5% CO_2_. Routine monitoring for morphology and contamination was performed.

For siRNA or plasmid transfection, cells were seeded in 6‐well plates (3–5 × 10^5^ cells per well) and transfected at ~80%–90% confluence using Lipofectamine 2000 (11668019, Thermo Fisher Scientific, MA, USA) according to the manufacturer's protocol. For siRNA transfection, 100 pmol of siRNA was used per well, while 2 μg of plasmid DNA was used for plasmid transfection. Medium was replaced with fresh DMEM 6 h after transfection.

Cells transfected with the *LINC01021*‐overexpression (*LINC01021*‐OE) plasmid were harvested 6 d post‐transfection, while other conditions were collected after 3 d. siRNA sequences are listed in Table S1.

### 2.5 Transcriptome Data Processing

RNA sequencing (RNA‐seq) datasets (accession number: GSE63577) were obtained from the Gene Expression Omnibus (GEO) database (https://www.ncbi.nlm.nih.gov/geo/). These datasets include transcriptomic profiles of young and senescent fibroblast cell lines, including human foreskin fibroblasts (HFF), BJ foreskin fibroblasts (BJ), IMR‐90 fetal lung fibroblasts (IMR90), and MRC‐5 lung fibroblasts (MRC5). Differential expression analysis was conducted in RStudio (R 4.4.3) using the DESeq2 package. Genes significantly upregulated in senescent cells were defined as those with an adjusted *p* value < 0.05 and log_2_ Fold Change (log_2_FC) > 0.

### 2.6 Senescent Cell Induction

Senescent cells were induced by three methods: ionizing radiation (IR), doxorubicin (DOX) treatment, and replicative exhaustion, as previously described (Y. He et al. 2020). Low‐passage HELF and HDF fibroblasts (< 25 passages) were used as non‐senescent controls or for senescence induction. IR‐induced senescence was established by exposing cells at ~70% confluence to 15 Gy of ionizing radiation using a small animal radiation research platform (Xstrahl Inc., Camberley, UK). Cells were subcultured at a 1:3 ratio 3 d after irradiation, with full senescence typically observed ~7 d later. DOX‐induced senescence was established by treating cells with 250 nM DOX (HY‐15142, MCE, Shanghai, China) for 24 h. After treatment, the medium was replaced with standard DMEM, and cells were cultured for an additional 7 d. Replicative senescence was induced by serially passaging HELF cells until cell division ceased, typically after approximately 35 passages.

### 2.7 SA‐β‐Gal Staining

SA‐β‐Gal staining was performed using a Senescence β‐Galactosidase Staining Kit (C0602, Beyotime Biotechnology, Shanghai, China) following the manufacturer's instructions. Briefly, cells were seeded in six‐well plates and washed with PBS, fixed at room temperature for 7 min, and then incubated with the staining solution overnight at 37°C. The stained cells were imaged using a microscope (SMZ‐171‐BLED, Motic, Xiamen, China). Blue‐stained cells were considered senescent.

### 2.8 Quantitative Real‐Time PCR (qRT‐PCR)

Total RNA was isolated from cultured cells using TRIzol reagent (15596018, Invitrogen, CA, USA) according to the manufacturer's instructions. RNA concentration and purity were assessed spectrophotometrically. Equal amounts of RNA were reverse‐transcribed into cDNA using the HP All‐in‐One qRT Master Mix II Kit (RT203, YoungGen, Kunming, China). Quantitative PCR (qPCR) was performed using 2 × TSINGKE Master qPCR Mix (SYBR Green I) (TSE201, TSINGKE, Beijing, China) on a CFX Connect Real‐Time PCR System (Bio‐Rad, CA, USA), with *GAPDH* serving as the internal control. Relative gene expression levels were calculated using the 2^^−ΔΔCt^ method. Primer sequences used in this study are provided in Table S2.

### 2.9 Cell Counting

Cell counts were determined manually using a hemocytometer under a light microscope. Cell suspensions were mixed and diluted to an appropriate concentration for counting. An aliquot of the diluted sample was loaded onto a hemocytometer, and cells were counted in duplicate at 100× magnification. The average number of cells per grid was used to calculate the final concentration using the standard hemocytometer formula.

### 2.10 EdU Assay

Cell proliferation was assessed using the Cell‐Light EdU Apollo567 In Vitro Kit (C10310‐1, RiboBio, Guangzhou, China) according to the manufacturer's instructions. Cells were seeded into 96‐well plates at a density of 3000 cells per well and allowed to adhere overnight. The cells were then incubated with the EdU working solution for 2 h, fixed with 4% paraformaldehyde for 15 min at room temperature, and permeabilized with 0.5% Triton X‐100. EdU incorporation was detected by staining with Apollo567, and nuclei were counterstained with DAPI. Fluorescence images were acquired using a fluorescence microscope.

### 2.11 Western Blot

Cells were washed twice with cold 1 × phosphate‐buffered saline (PBS; B540627‐0500, Sangon Biotech, Shanghai, China) and lysed on ice in RIPA buffer (BP‐115DG, Boston BioProducts, MA, USA) supplemented with PMSF (ST507, Beyotime Biotechnology, Shanghai, China) for 30 min. The lysates were sonicated at 35 W for 10 s, followed by centrifugation at 13,000 × g for 15 min at 4°C to remove insoluble debris. Protein concentrations were determined using a BCA Protein Assay Kit (P0010, Beyotime Biotechnology, Shanghai, China). Equal amounts of protein were mixed with 5 × Omni‐Easy Instant SDS‐PAGE Sample Buffer (LT101, EpiZyme, Shanghai, China), denatured at 95°C for 5–10 min, and separated by SDS‐PAGE on 12% polyacrylamide gels. Proteins were transferred onto 0.22‐μm PVDF membranes (Millipore, Bedford, MA, USA) using a wet transfer system (Bio‐Rad, CA, USA). Membranes were blocked with 5% (w/v) skim milk in TBS containing 0.1% Tween‐20 (TBS‐T) for 2 h at room temperature and incubated with primary antibodies overnight at 4°C. After washing with TBS‐T, membranes were incubated with HRP‐conjugated secondary antibodies for 1 h at room temperature. Protein bands were visualized using the SCG‐W3000 Plus chemiluminescence imaging system (Servicebio, Wuhan, China) and quantified with ImageJ software (NIH, MD, USA). All antibodies used in this study are listed in Table S3.

### 2.12 Plasmid Construction

To generate overexpression plasmids, the full‐length sequences of *LINC01021*, *RPL23A*, *SKP1*, *EPB41L2*, and *DAZAP1* were individually cloned into the pCDH‐CMV‐MCS‐EF1‐puro‐3×FLAG‐3×HA vector.

### 2.13 RNA Pull‐down and Mass Spectrometry (MS) Analysis

RNA was transcribed in vitro using T7 RNA polymerase (K102A01, Perfect mRNA, Hangzhou, China) and labeled at the 3′‐end with biotin using the Pierce RNA 3′ End Desthiobiotinylation Kit (20163, Thermo Fisher Scientific, MA, USA), according to the manufacturer's protocol. RNA pull‐down assays were conducted as previously described (P. Wang et al. 2014). Briefly, 50 pmol of biotin‐labeled RNA was refolded in RNA structure buffer (10 mM Tris, pH 7.0; 100 mM KCl; 10 mM MgCl_2_) by heating at 85°C for 5 min and snap‐cooling on ice. RNA‐protein complexes were captured using the Pierce Magnetic RNA‐Protein Pull‐Down Kit (20164, Thermo Fisher Scientific, MA, USA), washed, eluted, and denatured in 5 × SDS loading buffer. The recovered proteins were separated by gradient SDS‐PAGE and analyzed by MS for protein identification.

### 2.14 Chromatin Immunoprecipitation (ChIP)‐qPCR

*LINC01021*‐OE HELFs were crosslinked by incubating with 1% formaldehyde for 5 min at 37°C, followed by quenching with 0.125 M glycine for 10 min at room temperature. Cells were then washed twice with ice‐cold PBS containing PMSF and lysed using SDS lysis buffer from the ChIP Assay Kit (P2078, Beyotime Biotechnology, Shanghai, China). Chromatin was fragmented using a Bioruptor Plus sonicator (Diagenode, Seraing, Belgium) at 40 W for 30 cycles (10 s on/10 s off) at 4°C. The sheared chromatin extracts were precleared with Protein A/G agarose beads pre‐blocked with salmon sperm DNA for 30 min at 4°C. After removing 2% of the input as a control, the lysates were incubated overnight at 4°C with either an anti‐RBMX antibody (F1965, Selleckchem, TX, USA) or a control IgG (A7016, Beyotime Biotechnology, Shanghai, China). Immune complexes were captured with Protein A/G agarose beads for 2 h at 4°C and washed sequentially with low‐salt, high‐salt, LiCl, and TE buffers. DNA–protein complexes were eluted with 1% SDS / 0.1 M NaHCO_3_ and decrosslinked by incubation with 0.2 M NaCl at 65°C for 4 h. The samples were treated with proteinase K at 45°C for 1 h, and the purified DNA was isolated using a commercial DNA purification kit (D6492, OMEGA BIO‐TEK, GA, USA). Quantified DNA was analyzed by qPCR, with primer sequences provided in Table S2.

### 2.15 Protein Half‐Life Assay

Protein stability was assessed as previously described (Q. Zhang et al. 2020). Cells transfected with the indicated plasmids were treated with 50 μg/mL cycloheximide (CHX; GC17198, GLPBIO, CA, USA) at the specified time points. At each time point, cells were harvested, lysed in RIPA buffer with protease inhibitors, and denatured in SDS loading buffer at 95°C for 5 min. Protein levels were analyzed by immunoblotting with the specified antibodies.

### 2.16 RNA Half‐Life Assay

Cells transfected with the indicated plasmids were treated with 10 μg/mL Actinomycin D (Act D; GC16866, GLPBIO, CA, USA) to inhibit transcription. Total RNA was extracted at the indicated time points, and RNA stability was assessed by RT‐qPCR using specific primers. Relative RNA levels at each time point were normalized to the 0 h time point and plotted to evaluate RNA decay.

### 2.17 Protein Degradation Pathway Analysis

HELFs were transfected with the *LINC01021*‐OE plasmid for 6 d to achieve robust overexpression. Cells were then treated with 1 μM MG132 (HY‐13259, MCE, Shanghai, China), 10 μM chloroquine (HY‐17589AR, MCE, Shanghai, China), or 5 μM Q‐VD‐OPh (HY‐12305, MCE, Shanghai, China) for 12 h before being harvested for Western blot analysis.

### 2.18 Animals

Transgenic mice with a knock‐in (KI) allele for *LINC01021*‐OE were obtained from Shanghai Model Organisms Center Inc. (Shanghai, China). These mice were generated by CRISPR/Cas9‐mediated homologous recombination, inserting a CAG‐*LINC01021*‐polyA (PA) cassette into the Rosa26 locus on a C57BL/6J background. The CAG promoter drives constitutive and ubiquitous expression of *LINC01021* across tissues, independent of endogenous regulatory elements (Hasegawa et al. 2013). Briefly, Cas9 mRNA and guide RNA (gRNA) were synthesized by in vitro transcription, and a donor vector containing a 3.3 kb 5′ homology arm, the CAG‐*LINC01021*‐PA cassette, and a 3.3 kb 3′ homology arm was constructed via In‐Fusion cloning. Cas9 mRNA, gRNA, and donor vector were microinjected into C57BL/6J zygotes to generate F0 founders, which were confirmed by PCR and Sanger sequencing. These founders were backcrossed to wild‐type C57BL/6J mice to obtain heterozygous F1 progeny. All mice were maintained under specific pathogen‐free (SPF) conditions at the Kunming Institute of Zoology, Chinese Academy of Sciences. Animal experiments were approved by the Animal Ethics Committee of the Kunming Institute of Zoology, Chinese Academy of Sciences (IACUC‐RE‐2025‐08‐008) and conducted following the Guide for the Care and Use of Laboratory Animals.

### 2.19 Mouse Frailty Index (FI) Assessment

Mouse FI was assessed using a validated clinical deficit accumulation approach, as previously described (Feridooni et al. 2015). The FI comprised 23 health deficits spanning multiple physiological systems, including integument, musculoskeletal, auditory, ocular, digestive, urogenital, and body weight (see Table S4 for detailed scoring criteria). Each deficit was scored as 0 (absent), 0.5 (mild), or 1 (severe) according to predefined criteria. Body weight was recorded as a continuous numerical variable for each mouse. Forelimb grip strength was measured three times using a grip strength meter, and the mean value was used for analysis. These measurements were reported as raw physiological readouts and were not converted into standard deviation‐based categorical scores. The FI for each mouse was calculated by summing the individual deficit scores and dividing by the total number of assessed items (23), yielding a final FI value ranging from 0 (no deficits) to 1 (all deficits present).

### 2.20 Behavioral Assessments

#### 2.20.1 Beam Walking Test

Motor coordination and balance were assessed using the beam walking test. Mice were trained to traverse a horizontal acrylic beam (1 cm width, 100 cm length) elevated 50 cm above the surface. During the test session, both the latency to traverse the full length of the beam and the number of paw slips were recorded. Each mouse performed three consecutive trials with a 10‐min intertrial interval, and the mean value was used for subsequent analysis.

#### 2.20.2 Rotarod Test

Motor performance and endurance were evaluated using an accelerating rotarod apparatus (SA102, SANS, Nanjing, China). On day 1, mice were habituated to the apparatus at a constant speed of 10 rpm for 10 min. On day 2, mice underwent training at 10 rpm for 5 min, followed by stepwise acceleration at 5 rpm every 2 min. During the test phase, mice were placed on the stationary rod, which accelerated from 4 rpm to 35 rpm over 4 min, including a 1‐min constant‐speed period at 4 rpm before acceleration. Each mouse completed three independent trials with 30‐min rest intervals. Latency to fall was recorded for each trial.

#### 2.20.3 Treadmill Fatigue Test

Exercise capacity was assessed using a motorized treadmill system (SA101, SANS, Nanjing, China). Mice were acclimated for three consecutive days at a speed of 5 m/min for 5 min per day without incline and with a mild electrical stimulus (0.3 mA). On the test day, mice were subjected to a 5‐degree incline and began running at 10 m/min for 10 min. The speed was subsequently increased by 2 m/min every 5 min until exhaustion, defined as remaining on the shock grid for more than 5 s without resuming running.

#### 2.20.4 Pole Test

The pole test was performed to assess bradykinesia and motor coordination. Mice were placed head‐upward on the top of a rough wooden pole (50 cm height, 8 mm diameter, 30‐degree inclination). The time required to turn downward and the total time to descend to the base were recorded. Mice underwent one day of training followed by three test trials (maximum duration of 60 s per trial, with 30‐min intertrial intervals). The average of the three trials was used for statistical analysis.

### 2.21 In Vivo Senescence Imaging

In vivo senescence imaging was performed using the Senescence‐Tracker Near‐Infrared (NIR) probe (C0603, Beyotime Biotechnology, Shanghai, China), as previously described (Hu et al. 2023). The probe was dissolved in DMSO to generate a 10 mM stock solution and diluted in 1% DMSO, 2% Tween‐80, and 97% sterile saline to a final concentration of 5 μM. Mice received a single intravenous tail‐vein injection of 100 μL probe solution. Whole‐body fluorescence images were acquired 24 h post‐injection using an IVIS Lumina 3 imaging system (PerkinElmer, CT, USA) under identical acquisition settings. Regions of interest (ROIs) were manually defined over each mouse, and fluorescence intensity was quantified as total radiant efficiency using Living Image Software v4.4. All imaging and analysis parameters were kept constant across experimental groups.

### 2.22 Statistical Analysis

Data are presented as mean ± standard error of the mean (SEM). Comparisons between two groups were performed using an unpaired two‐tailed Student's *t*‐test, with Welch's correction applied when variances were unequal. Comparisons among more than two groups were conducted using one‐way analysis of variance (ANOVA). A *p*‐value < 0.05 was considered statistically significant (* *p* < 0.05, ** *p* < 0.01, *** *p* < 0.001; ns, not significant).

## 3 Results

### 3.1 Primate‐Conserved lncRNAs Exhibit Widespread and Recurrent Associations With Aging Across Human Tissues

**FIGURE 1:**
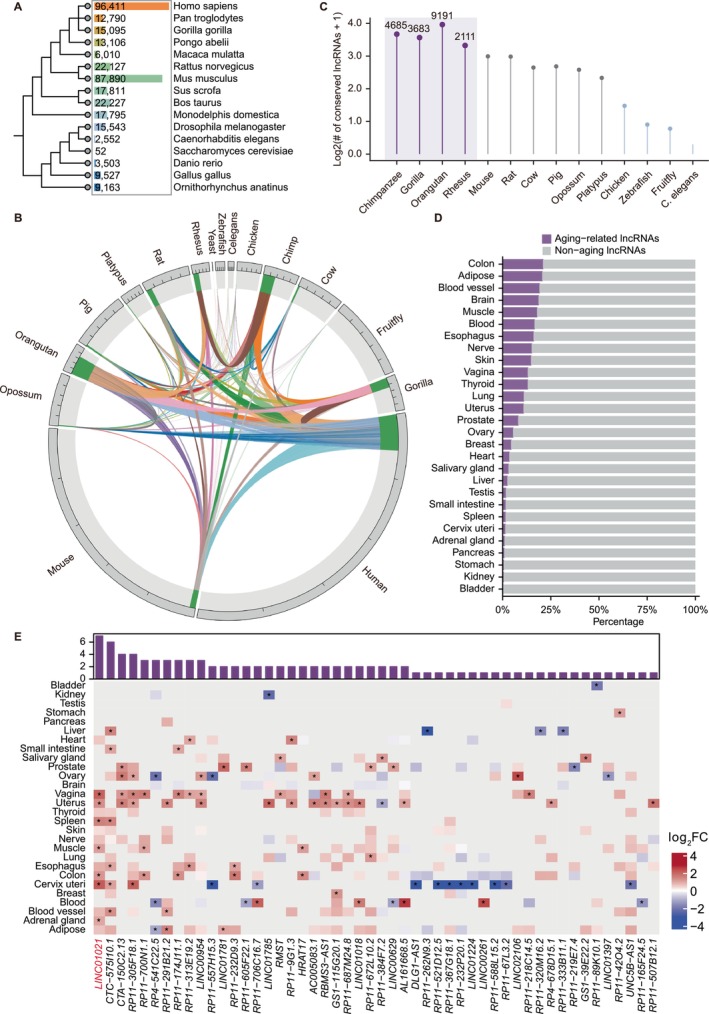
Evolutionary distribution and conservation of lncRNAs across species. (A) Phylogenetic tree of representative species annotated with the number of lncRNAs identified in each genome. (B) Circular visualization of lncRNA conservation across species, with links indicating shared lncRNAs between species pairs. (C) Relative enrichment of conserved human lncRNAs in primates compared with non‐primate mammals. (D) Distribution of primate‐conserved lncRNAs exhibiting age‐associated expression across human tissues. (E) Heatmap showing age‐associated expression changes of primate‐conserved lncRNAs across human tissues under stringent selection criteria (FDR < 0.05 and log_2_FC > 1). Color scale indicates log_2_FC with age (red, upregulation; blue, downregulation). The bar plot above summarizes the number of tissues in which each lncRNA exhibits significant age‐associated expression.

LncRNAs constitute a major class of regulatory transcripts in eukaryotic genomes but are characterized by rapid evolutionary turnover and pronounced lineage specificity (Mattick et al. 2023; Statello et al. 2021). To systematically assess the evolutionary distribution of lncRNAs, we compiled annotated lncRNA catalogs from the NONCODE database across multiple representative species. As shown in Figure 1A, the number of annotated lncRNAs varies dramatically among species, ranging from only 52 in yeast to more than 96,000 in humans. Notably, substantial discrepancies were also observed among closely related primates: chimpanzee, gorilla, and orangutan each harbor approximately 6,000–15,000 annotated lncRNAs, markedly fewer than reported in humans. These differences are unlikely to reflect true biological absence, but instead largely arise from variation in sequencing depth, annotation strategies, and research intensity, underscoring the limitations of absolute lncRNA counts for inferring functional relevance across species.

To move beyond catalog size and directly evaluate evolutionary relationships, we performed pairwise comparisons of lncRNAs across species to assess sequence conservation (Figure 1B). Consistent with prior observations, lncRNAs exhibited strong species specificity, with only a small fraction shared between any two species. Importantly, when human lncRNAs were used as the reference, a substantially larger fraction showed detectable conservation within primate species compared with non‐primate mammals. Based on this primate‐skewed conservation pattern, we identified a set of 7098 human lncRNAs that are conserved in at least one other primate species but absent from non‐primate species, hereafter referred to as primate‐conserved lncRNAs (Figure 1C). These transcripts combine evolutionary constraints with lineage restriction, suggesting that they may encode regulatory functions preferentially relevant to primate biology.

To investigate whether primate‐conserved lncRNAs are implicated in aging, we analyzed their age‐associated expression patterns across multiple human tissues using GAMs. Across all tissues examined, a subset of primate‐conserved lncRNAs exhibited significant associations with chronological age (Figure 1D), with the colon displaying the largest number of aging‐associated candidates. Integrative analysis further revealed that many of these lncRNAs showed age‐dependent expression changes across multiple tissues, with some exhibiting significant associations in more than 16 tissues (Figure S1A), indicating potential roles in systemic or organism‐wide aging regulation.

To define a high‐confidence set of aging‐associated candidates, we applied more stringent criteria requiring both statistical significance (FDR < 0.05) and substantial age‐dependent expression changes (log_2_FC > 1). This analysis identified a core cohort of primate‐conserved lncRNAs with robust and recurrent age‐associated expression across tissues (Figure 1E), such as *LINC01021*, *CTC‐575 l10.1*, *CTA‐150C2.13*, *RP11‐305F18.1*, *RP11‐700 N1.1*, *RP4‐541C22.5*, and *RP11‐291B21.2*. Among these, *LINC01021* emerged as the most broadly associated transcript, exhibiting significant age‐related expression changes across seven tissues, including the adrenal gland, cervix uteri, and colon (Figure 1E). In addition to *LINC01021*, several other primate‐conserved lncRNAs, such as *CTC‐575 l10.1*, *CTA‐150C2.13*, and *RP11‐305F18.1*, also displayed consistent age‐associated expression patterns across multiple tissues (Figure 1E). Together, these findings position *LINC01021* as a representative exemplar of a broader class of aging‐associated primate‐conserved lncRNAs rather than an isolated outlier. Based on its breadth of age association, recurrent induction across independent aging contexts, and suitability for functional interrogation, *LINC01021* was selected for subsequent mechanistic investigation.

### 3.2 *LINC01021* Is Robustly Upregulated Across Multiple Senescence Models in Human Fibroblasts

**FIGURE 2:**
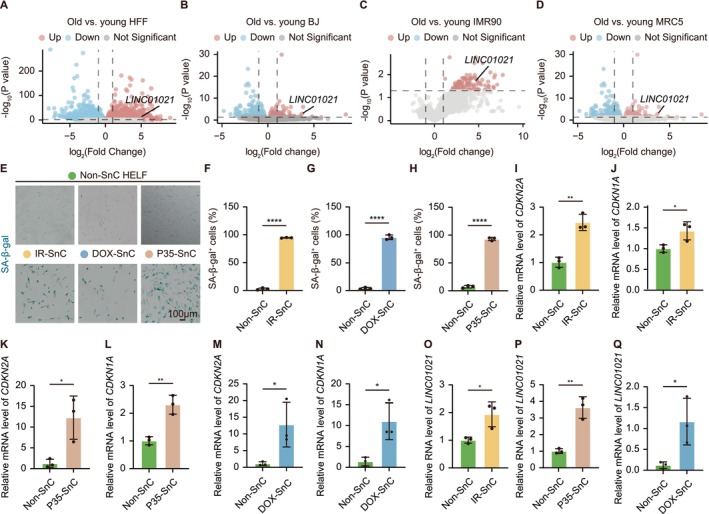
*LINC01021* is robustly upregulated across multiple senescence models in human fibroblasts. (A‐D) Volcano plots showing transcriptional changes between young and senescent fibroblasts in four independent human fibroblast strains (HFF, BJ, IMR90, and MRC5), highlighting consistent upregulation of *LINC01021*. (E) Representative SA‐β‐Gal staining images of HELF cells subjected to IR‐induced, DOX‐induced, or replicative senescence. (F‐H) Quantification of SA‐β‐Gal^+^ cells corresponding to the conditions shown in (E). (I‐N) qRT‐PCR analysis of senescence markers *CDKN2A* and *CDKN1A* in HELF cells under IR‐induced (I and J), replicative (K and L), or DOX‐induced (M and N) senescence. (O‐Q) qRT‐PCR analysis showing induction of *LINC01021* expression in HELF cells across the indicated senescence models. Data are shown as mean ± SEM from at least three independent experiments.

Following the identification of primate‐conserved lncRNAs with recurrent age‐associated expression across human tissues, we next examined whether *LINC01021*, as a representative candidate, exhibits consistent induction in established human cellular models of senescence. We first analyzed a publicly available RNA‐seq dataset comprising four human fibroblast strains (HFF, BJ, IMR90, and MRC5) subjected to either replicative or stress‐induced senescence (Marthandan et al. 2016). Across these well‐established models, which recapitulate core features of cellular aging, *LINC01021* emerged as a robust aging‐associated primate‐conserved lncRNA that was reproducibly upregulated across all four fibroblast strains under both senescence paradigms (Figure 2A–D).

To experimentally validate these observations, we established three independent senescence models in HELFs, including IR‐induced senescence, DOX‐induced senescence, and replicative senescence induced by serial passaging. Successful induction of senescence was confirmed by a marked increase in SA‐β‐Gal positive cells (Figure 2E–H) and significant upregulation of canonical senescence regulators *CDKN2A* and *CDKN1A* at the mRNA level (Figure 2I–N). Consistent with the bioinformatic analyses, qRT‐PCR revealed a robust and highly reproducible increase in *LINC01021* expression across all three senescence models (Figure 2O–Q). Notably, the magnitude of *LINC01021* induction was comparable to, and in some conditions exceeded that of *CDKN2A* and *CDKN1A*, further supporting its tight association with the senescent state.

### 3.3 *LINC01021* Modulates Cellular Senescence in Human Fibroblasts

**FIGURE 3:**
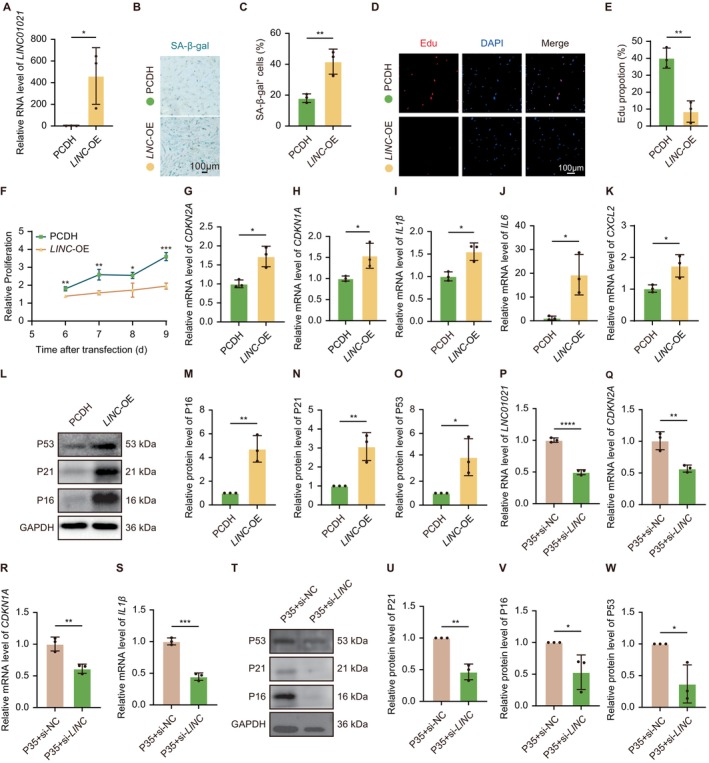
*LINC01021* modulates cellular senescence in human fibroblasts. (A) qPCR analysis confirming *LINC01021* overexpression in HELF cells. (B, C) Representative SA‐β‐Gal staining and quantification showing increased senescence upon *LINC01021* overexpression. (D, E) Representative EdU staining and quantification indicating reduced DNA synthesis in *LINC01021*‐OE HELF cells. (F) Proliferation curves comparing *LINC01021*‐OE HELF cells with vector controls. (G‐K) qRT‐PCR of *CDKN2A*, *CDKN1A*, and SASP‐associated genes in *LINC01021*‐OE HELF cells. (L‐O) Western blot analysis and quantification of P16, P21, and P53 in *LINC01021*‐OE HELF cells. (P‐S) qRT‐PCR analysis of *LINC01021*, *CDKN2A*, *CDKN1A*, and SASP‐associated genes in replicative senescent HELF cells following *LINC01021* knockdown. (T‐W) Western blot analysis and quantification of P21, P16, and P53 in replicative senescent HELF cells after *LINC01021* knockdown. Data are shown as mean ± SEM from at least three independent experiments. SASP, senescence‐associated secretory phenotype.

Given the consistent and reproducible induction of *LINC01021* across multiple cellular senescence models, we next investigated whether *LINC01021* functionally contributes to the establishment of senescent phenotypes in human fibroblasts. Gain‐of‐function experiments were first performed in HELFs, in which overexpression of *LINC01021* was confirmed by qRT‐PCR (Figure 3A). Compared with control cells, *LINC01021*‐OE HELFs exhibited a significantly increased proportion of SA‐β‐Gal positive cells, a reduced fraction of EdU‐positive proliferating cells, and an overall impairment of proliferative capacity, as reflected by slower cell growth rates (Figure 3B–F). Consistent phenotypic effects were also observed in HDFs, in which *LINC01021*‐OE similarly increased SA‐β‐Gal positivity and reduced EdU incorporation (Figure S2A–D), indicating that the pro‐senescent effects of *LINC01021* are not restricted to a single fibroblast lineage.

At the molecular level, *LINC01021* overexpression induced the expression of core senescence effectors, including *CDKN2A* and *CDKN1A*, and promoted the transcription of multiple senescence‐associated secretory phenotype (SASP) factors, such as *IL1β*, *IL6*, and *CXCL2* (Figure 3G–K). Concordantly, protein levels of P16 and P21 were markedly increased following *LINC01021* overexpression (Figure 3L–N). Notably, P53 protein abundance was also elevated under these conditions (Figure 3L,O), a pattern that contrasts with previous reports describing a suppressive role of *LINC01021* on P53 activity in cancer cells, highlighting potential context‐dependent regulation (Han et al. 2021).

To complement these gain‐of‐function analyses, loss‐of‐function experiments were performed across three independent senescence models. Knockdown of *LINC01021* consistently reduced the expression of *CDKN2A* and *CDKN1A* and attenuated the induction of SASP‐associated genes (Figures 3P–S and S2E–R). At the protein level, P16 and P21 were correspondingly decreased across all senescence models upon *LINC01021* depletion, whereas P53 protein levels decreased upon knockdown and increased upon overexpression, remaining positively correlated with *LINC01021* expression (Figures 3T–W and S2S–Z). Together, these gain‐ and loss‐of‐function analyses demonstrate that *LINC01021* actively modulates key cellular senescence features in human fibroblasts and reveal a context‐dependent relationship between *LINC01021* and P53 regulation that differs from its previously reported role in cancer cells (Kaller et al. 2024; Li et al. 2017).

### 3.4 RBMX Mediates *LINC01021* ‐Dependent Senescence Regulation

**FIGURE 4:**
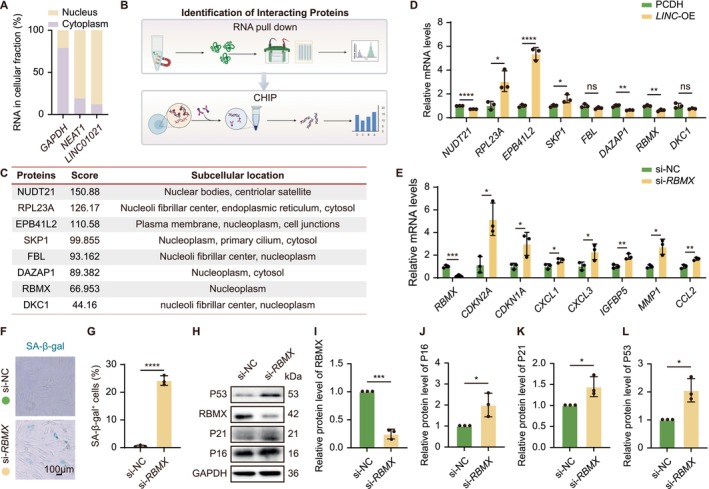
RBMX is a key mediator of *LINC01021*‐dependent senescence regulation. (A) Subcellular fractionation showing predominant nuclear localization of *LINC01021*. (B) Schematic overview of the workflow for identifying and validating *LINC01021*‐interacting proteins, including RNA pull‐down coupled with mass spectrometry (MS) and subsequent chromatin immunoprecipitation (ChIP) assays. (C) Candidate nuclear proteins identified by *LINC01021* RNA pull‐down and MS. (D) qRT‐PCR analysis of selected candidate mRNAs in *LINC01021*‐OE HELF cells. (E) qRT‐PCR analysis of *RBMX*, *CDKN2A*, *CDKN1A*, and SASP‐associated genes in si‐*RBMX* HELF cells. (F, G) Representative SA‐β‐Gal staining and quantification after *RBMX* knockdown. (H‐L) Western blot analysis and quantification of RBMX, P16, P21, and P53 in si‐*RBMX* HELF cells. Data are shown as mean ± SEM from at least three independent experiments.

To explore the molecular mechanism underlying *LINC01021*‐mediated senescence regulation, particularly its impact on P53‐associated pathways, we first examined the subcellular localization of *LINC01021*. Subcellular fractionation analyses revealed that *LINC01021* is predominantly enriched in the nucleus (Figure 4A), prompting us to focus on nuclear‐associated proteins in subsequent mechanistic investigations. We therefore performed RNA pull‐down coupled with MS to identify *LINC01021*‐interacting proteins, which yielded 8 candidate binding partners with annotated nuclear localization (Figure 4B,C).

To assess the functional relevance of these candidates in *LINC01021*‐associated senescence, we examined their expression changes in *LINC01021*‐OE HELFs. Among the 8 candidates, the mRNA levels of genes encoding 3 candidate proteins (NUDT21, DAZAP1, and RBMX) were negatively correlated with *LINC01021* expression, 3 (RPL23A, EPB41L2, and SKP1) were positively correlated, and 2 (FBL and DKC1) showed no significant changes (Figure 4D). To functionally interrogate their roles, negatively correlated candidates were individually depleted, whereas positively correlated candidates were ectopically expressed in HELFs. Notably, knockdown of *RBMX*, but not *NUDT21* or *DAZAP1*, resulted in robust induction of the senescence regulators *CDKN2A* and *CDKN1A*, along with multiple SASP genes, including *CXCL1*, *CXCL3*, *IGFBP5*, *MMP1*, and *CCL2* (Figures 4E and S3A,B). In contrast, overexpression of *RPL23A*, *EPB41L2*, or *SKP1* failed to elicit comparable senescence‐associated transcriptional responses (Figure S3C–E) indicating RBMX as the primary functional mediator among the identified *LINC01021*‐interacting candidates.

Consistent with these transcriptional changes, *RBMX* knockdown markedly increased the proportion of SA‐β‐Gal‐positive HELFs and elevated protein levels of the senescence effectors P16 and P21 (Figure 4F–K). Notably, *RBMX* knockdown also led to an increase in P53 protein (Figure 4H,L), closely mirroring the molecular phenotype observed upon *LINC01021* overexpression. To assess P53 dependency, we knocked down *p53* via siRNA in *LINC01021*‐overexpressing cells and examined P53/P21 pathway activity. *p53* depletion markedly attenuated *LINC01021*‐induced P21 activation (Figure S3F,G) indicating that *LINC01021* promotes senescence at least in part through P53‐dependent signaling downstream of RBMX. Together, these findings position RBMX as a key effector downstream of *LINC01021* that mediates senescence regulation via P53, and underscore the essential role of P53 in this pathway.

### 3.5 *LINC01021* Regulates RBMX Abundance Predominantly Through DAZAP1‐Dependent Post‐Transcriptional Mechanisms

**FIGURE 5:**
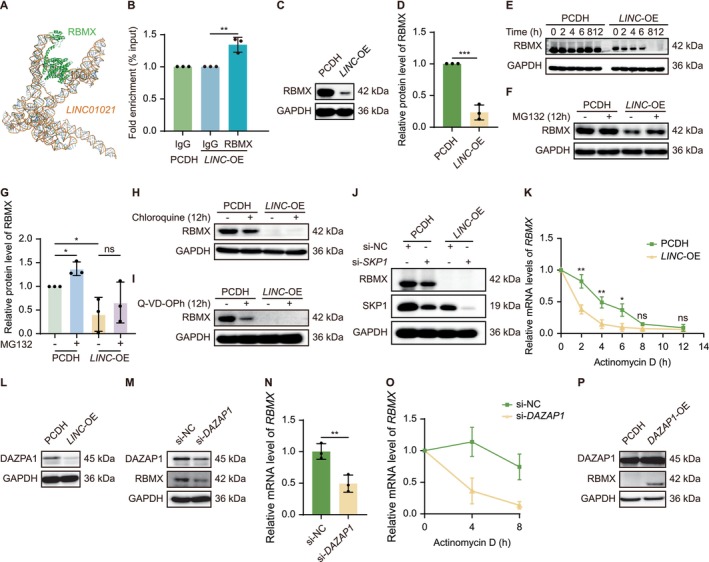
*LINC01021* regulates RBMX abundance predominantly through DAZAP1‐dependent post‐transcriptional mechanisms. (A) AlphaFold‐based modeling predicting a potential structural interface between *LINC01021* and RBMX. (B) ChIP‐qPCR analysis showing low but reproducible chromatin‐associated enrichment of *LINC01021* in RBMX immunoprecipitates. (C, D) Western blot analysis and quantification of RBMX protein levels in *LINC01021*‐OE HELF cells. (E) Cycloheximide chase analysis assessing RBMX protein stability upon *LINC01021* overexpression. (F, G) MG132 treatment partially restores RBMX protein levels in *LINC01021*‐OE HELF cells. (H‐J) Inhibition of lysosomal degradation (chloroquine), caspase‐dependent proteolysis (Q‐VD‐OPh), or SCF E3 ligase component SKP1 does not rescue RBMX protein levels. (K) Actinomycin D chase analysis showing accelerated *RBMX* mRNA decay in *LINC01021*‐OE HELF cells. (L) Western blot analysis of DAZAP1 protein levels in *LINC01021*‐OE HELF cells. (M) RBMX protein levels following *DAZAP1* knockdown. (N, O) qRT‐PCR and mRNA stability analyses demonstrating DAZAP1‐dependent regulation of *RBMX* transcript abundance. (P) RBMX protein levels following *DAZAP1* overexpression. Data are shown as mean ± SEM from at least three independent experiments.

Given that RBMX functions as a key downstream mediator of *LINC01021*‐induced cellular senescence, we next investigated how *LINC01021* regulates RBMX abundance. AlphaFold‐based structural modeling predicted a putative three‐dimensional association between *LINC01021* and RBMX (Figure 5A); however, ChIP‐qPCR using an anti‐RBMX antibody revealed only low but reproducible enrichment of *LINC01021* (Figures 4B and 5B), suggesting an indirect chromatin‐associated relationship rather than a stable RNA–protein interaction. Consistent with a regulatory effect, *LINC01021* overexpression in HELFs dramatically reduced RBMX protein levels (Figure 5C,D) and shortened the RBMX protein half‐life (Figure 5E), indicating that *LINC01021* negatively regulates RBMX abundance.

To assess whether RBMX destabilization involves proteolytic pathways, we examined the major mechanisms of protein degradation. Pharmacological inhibition of the proteasome using MG132 marginally restored RBMX protein levels in *LINC01021*‐OE cells (Figure 5F,G), whereas inhibition of lysosomal degradation or caspase‐dependent proteolysis had no detectable effect (Figures 5H,I and S4A,B). SKP1, a core adaptor component of SCF (SKP1‐CUL1‐F‐box) E3 ubiquitin ligase complexes that mediate proteasomal protein turnover (Zeng et al. 2025), was identified among candidate *LINC01021*‐interacting proteins in the RNA pull‐down assay; however, *SKP1* knockdown failed to rescue RBMX protein levels (Figures 5J and S4C), indicating that proteasome‐mediated degradation contributes weakly and in an SKP1‐independent manner. In parallel, qPCR analysis revealed that *LINC01021* overexpression significantly reduced *RBMX* mRNA levels (Figure 4D), prompting us to examine post‐transcriptional regulation.

Act D chase experiments demonstrated that *LINC01021* markedly accelerated *RBMX* mRNA decay (Figure 5K), indicating a substantial contribution of mRNA destabilization to *RBMX* downregulation. Among proteins identified in the RNA pull‐down screen, the RNA‐binding protein DAZAP1, previously implicated in mRNA stability control (Qiu, Li, et al. 2025; Wang et al. 2021; Zhang et al. 2024), was significantly reduced at both mRNA and protein levels upon *LINC01021* overexpression (Figure 4D; Figures 5L and S4D). Functionally, *DAZAP1* depletion decreased RBMX protein and mRNA levels and accelerated RBMX transcript decay (Figures 5M–O and S4E,F), whereas *DAZAP1* overexpression robustly increased RBMX protein abundance (Figures 5P and S4G,H). Together, these findings indicate that *LINC01021* suppresses RBMX expression predominantly through a DAZAP1‐dependent post‐transcriptional mechanism governing *RBMX* mRNA stability, with a limited contribution from proteasome‐mediated protein turnover.

### 3.6 *LINC01021* Overexpression Promotes Aging‐Like Phenotypes in Humanized Transgenic Mice

**FIGURE 6:**
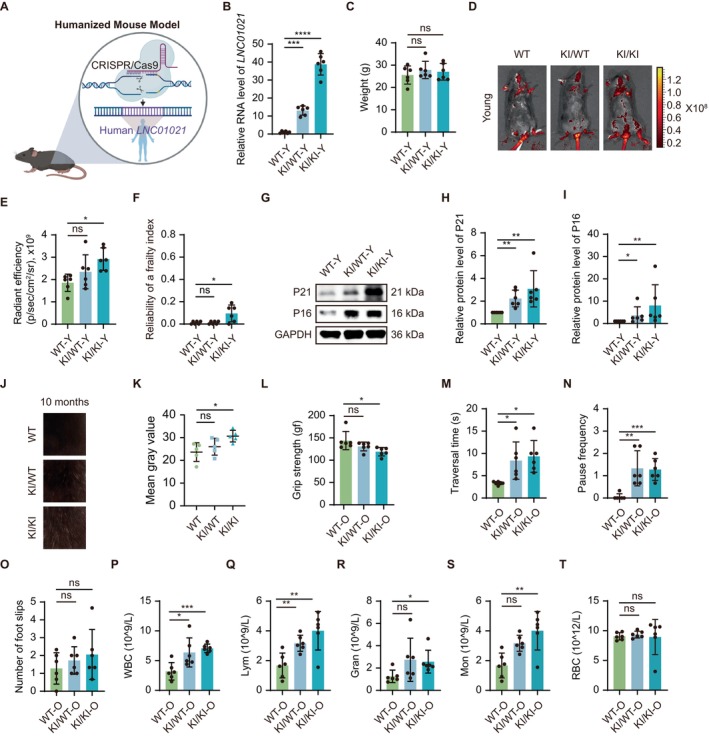
*LINC01021* overexpression promotes aging‐like phenotypes in humanized transgenic mice. (A) Schematic of the humanized *LINC01021*‐KI mouse model. (B) qRT‐PCR analysis of human *LINC01021* expression in spleen from young WT, KI/WT, and KI/KI mice. (C) Body weight of young mice. (D, E) Representative NIR fluorescence imaging using a SA‐β‐Gal responsive probe and quantification of senescence‐associated signal intensity in young mice. (F) Frailty index in young mice. (G‐I) Western blot analysis and quantification of P21 and P16 protein levels in spleen from young mice. (J, K) Representative images and quantification of fur depigmentation in 10‐month‐old male mice. (L) Forelimb grip strength in aged mice. (M‐O) Balance and coordination performance in aged mice assessed by balance beam traversal time (M), pause frequency (N), and foot slips (O). (P–T) Hematological parameters in aged mice, including white blood cells (WBC), lymphocytes, granulocytes, monocytes, and red blood cells (RBC).

To assess whether ectopic expression of the primate‐specific lncRNA *LINC01021* is sufficient to elicit aging‐associated phenotypes in vivo, we generated a humanized *LINC01021* KI mouse model using CRISPR‐Cas9. Correct transgene integration was confirmed by PCR genotyping, and *LINC01021* expression in the spleen was verified by qRT‐PCR (Figures 6A,B and S5A), as rodents lack an endogenous ortholog of this primate‐specific lncRNA. Body weight measurements revealed no significant differences between KI and WT mice at the examined ages, indicating that overall growth was not significantly affected (Figures 6C and S5B). To further quantify senescence burden in vivo, we applied near‐infrared senescence imaging using the activatable near‐infrared β‐galactosidase probe, a β‐galactosidase‐responsive fluorescent sensor that emits in the NIR spectrum upon enzymatic cleavage by SA‐β‐Gal, enabling real‐time detection of senescent cells in deep tissues (Hu et al. 2023). This analysis revealed a significantly elevated senescence signal in young adult KI/KI mice compared with WT controls, whereas aged KI mice exhibited a trend toward increased signal intensity that did not reach statistical significance (Figures 6D,E and S5C,D), suggesting that *LINC01021* expression may contribute to the onset of cellular senescence in vivo.

Consistent with increased senescence burden, comprehensive phenotypic analyses uncovered signs of accelerated functional decline in *LINC01021*‐expressing mice. Frailty indices increased more steeply in young KI/KI animals relative to WT littermates, while aged transgenic mice displayed a similar upward trend without statistical significance, likely reflecting increased inter‐individual variability (Figures 6F and S5E). In parallel, both young and aged KI mice showed a tendency toward reduced whisker number compared with WT controls (Figure S5F,G). At the molecular level, both heterozygous (KI/WT) and homozygous (KI/KI) young mice displayed marked upregulation of the senescence markers P16 and P21 in the spleen (Figure 6G–I), indicating that ectopic *LINC01021* expression enhances senescence‐associated gene expression in vivo, thereby linking organismal phenotypes to underlying cellular senescence processes.

In addition, male transgenic mice exhibited early‐onset coat depigmentation beginning around 8 months of age, with homozygous KI/KI animals developing pronounced whitening by 10 months (Figure 6J,K), further supporting premature aging‐associated phenotypes.

Functional assessments further revealed aging‐related impairments in motor performance and systemic immune homeostasis. From 10 months of age onward, transgenic mice exhibited reduced grip strength compared with WT controls, with the most pronounced decline observed in KI/KI mice compared to their WT counterparts, while younger KI mice showed a similar downward trend (Figures 6L and S5H). In beam‐walking assays, aged transgenic mice required more time to traverse the beam and paused more frequently, accompanied by a trend toward increased foot slips (Figures 6M–O and S5I–K). In contrast, rotarod and treadmill performance did not differ significantly between WT and KI mice (Figure S5L–S), suggesting that motor deficits associated with *LINC01021* expression are task‐specific rather than globally impaired. Hematological analyses revealed systemic aging‐associated immune alterations, including increased white blood cell and lymphocyte counts in both KI genotypes (Figures 6P,Q and S5T,U), with additional elevations in granulocytes and monocytes in KI/KI mice (Figures 6R,S and S5V,W), while platelet and red blood cell counts remained unchanged (Figures 6T and S5X–Z).

Collectively, these findings demonstrate that ectopic expression of human *LINC01021* contributes to multiple aging‐like phenotypes in vivo, linking primate‐specific *LINC01021* activity to organismal senescence and functional decline.

## 4 Discussion

Aging is shaped by conserved molecular hallmarks yet unfolds along distinct trajectories across species, implying the existence of lineage‐restricted regulatory layers that modulate canonical aging programs (López‐Otín et al. 2023). Here, through comparative evolutionary analyses integrated with cross‐species aging‐associated transcriptomic profiling, we identify a set of primate‐specific lncRNAs that exhibit recurrent age‐dependent expression patterns. Functional interrogation across multiple senescence paradigms and a humanized KI mouse model demonstrates that lineage‐specific lncRNAs can engage conserved senescence machinery and influence aging phenotypes. Using *LINC01021* as a representative example, we further delineate a mechanism by which this primate‐specific lncRNA, through interaction with the RNA‐binding protein DAZAP1, destabilizes RBMX, ultimately activating the P53 pathway and translating lineage‐specific regulatory information into measurable cellular and organismal aging outcomes. Together, these findings provide causal evidence that non‐coding regulatory novelty contributes to lineage‐restricted aging phenotypes.

A key conceptual contribution of this study is the establishment of an evolutionary framework for prioritizing aging‐associated lncRNAs, in which lineage restriction is leveraged as an informative biological signal rather than treated as a technical limitation. By systematically integrating primate‐specific conservation with age‐dependent expression across multiple human tissues, we uncovered a substantial subset of lncRNAs exhibiting robust and recurrent associations with aging. The broad tissue distribution of these associations, spanning reproductive, immune, muscular, gastrointestinal, and endocrine systems, suggests that primate‐specific lncRNAs are not confined to tissue‐specific contexts but may participate in higher‐order, systemic aging programs that operate across physiological axes most vulnerable to age‐related decline (Marttila et al. 2020; Sherazi et al. 2023). Importantly, the absence of orthologs for many of these lncRNAs in rodents highlights an inherent gap in conventional mammalian aging models and raises the possibility that certain dimensions of human aging are governed by regulatory layers that emerged relatively late in evolution (Mattick et al. 2023; Yue et al. 2014). In this context, primate‐conserved lncRNAs may serve as molecular interfaces through which conserved stress‐response pathways are fine‐tuned in a lineage‐dependent manner, providing a plausible explanation for how shared aging hallmarks give rise to divergent organismal aging trajectories across species (López‐Otín et al. 2023; Mattick et al. 2023; Sherazi et al. 2023).

Extending this evolutionary framework into the context of cellular aging, our findings identify *LINC01021* as a representative primate‐specific lncRNA that directly interfaces with the core senescence regulatory network in human fibroblasts. Its consistent upregulation across replicative, IR‐induced, and DOX‐induced senescence models indicates that *LINC01021* responds to convergent aging signals rather than to a single category of cellular insult. This pattern is in line with independent reports linking *LINC01021* to senescence‐associated states in human cells (Frediani et al. 2025; Rossi et al. 2023; Wang et al. 2025), supporting its relevance as a bona fide regulator of cellular aging. Functionally, elevated *LINC01021* expression reinforces growth arrest and amplifies senescence‐associated outputs. Notably, the role of *LINC01021* is highly context‐dependent. In proliferative cancer cells, it exerts oncogenic effects primarily by attenuating P53 responses via MDM2‐related pathways, thereby promoting cell cycle progression and tumor development (Kaller et al. 2024; Kaller et al. 2017; Li et al. 2017; Malakar 2024). In contrast, in senescent fibroblasts, *LINC01021* activates rather than suppresses the P53 pathway through the DAZAP1‐RBMX axis, thereby reinforcing cellular senescence. This switch from P53 inhibition in cancer to P53 activation in senescence exemplifies context‐dependent lncRNA regulation, which may arise from differences in cellular state, RNA secondary structure, or protein and epigenetic interactions. Further studies are warranted to elucidate the molecular basis of this switch, which could reflect an evolutionary adaptation in primates for fine‐tuning regulatory pathways according to distinct physiological demands, such as proliferation versus long‐term tissue maintenance.

Mechanistically, our study identifies RBMX as a critical regulatory node through which primate‐specific lncRNAs interface with conserved senescence pathways. RBMX is a multifunctional RNA‐binding protein implicated in RNA processing, genome stability, and transcriptional restraint of P53‐responsive programs (Cai et al. 2021; He et al. 2024), positioning it as a gatekeeper of stress‐induced growth arrest. We show that age‐associated upregulation of *LINC01021* leads to a dramatic reduction in RBMX abundance, thereby releasing RBMX‐mediated constraint on P53 signaling and lowering the threshold for senescence induction. Notably, this regulation does not occur through enhanced proteasomal degradation but instead through accelerated decay of *RBMX* mRNA, highlighting post‐transcriptional control as a key interface between lncRNAs and aging pathways (Li et al. 2023; Mattick et al. 2023). Our data further indicate that *LINC01021* disrupts DAZAP1‐mediated stabilization of *RBMX* transcripts, providing a mechanistic link between lncRNA scaffolding functions and RNA‐binding protein networks. Although DAZAP1 itself has not been extensively characterized as an aging regulator, its established role in RNA metabolism places it within a class of RNA‐binding proteins that are increasingly recognized as vulnerable nodes in aging‐associated decline (Choudhury et al. 2014; Conlon and Manley 2017; Di Fraia et al. 2025; Yang et al. 2009). This RNA‐centric and modular mode of regulation may therefore represent a common strategy through which evolutionary innovation shapes lineage‐specific aging phenotypes.

Importantly, the regulatory logic uncovered here aligns with a broader emerging framework in which lncRNAs act as integrative modulators of aging across cellular and tissue contexts. While many aging‐associated lncRNAs are evolutionarily conserved, comparing their regulatory magnitude and functional scope with primate‐specific lncRNAs provides important context for assessing lineage‐specific regulatory innovation. In skeletal muscle, age‐associated decline of the conserved lncRNA *MALAT1* enhances TGF‐β1 signaling and disrupts myoblast proliferation and cell‐cycle progression through P53‐dependent mechanisms, contributing to impaired regenerative capacity (Ruan et al. 2021). Conversely, in vascular smooth muscle cells undergoing replicative aging, *NEAT1* is robustly upregulated and reinforces senescence‐associated secretory programs, including IL8 expression, via P53‐centered feedback loops (Rossi et al. 2023). Notably, within these aging‐relevant contexts, the primate‐specific *LINC01021* exhibits even stronger induction than conserved lncRNAs, highlighting the potential for lineage‐restricted regulatory layers to exert disproportionate influence on aging pathways. Beyond cellular models, dysregulation of circulating lncRNAs such as *H19*, *NEAT1*, and *PVT1* correlates with sarcopenia, frailty, cardiovascular dysfunction, and neurodegenerative progression, suggesting that lncRNA‐mediated regulatory shifts are reflected at the organismal level (Aparicio et al. 2025). Together, these observations position lncRNAs as molecular conduits linking cell‐intrinsic senescence programs to tissue dysfunction and age‐related disease phenotypes, providing a broader context for understanding how primate‐specific lncRNAs such as *LINC01021* may shape human aging trajectories.

In a broader evolutionary context, lineage‐restricted regulatory innovations have increasingly been recognized as important modulators of species‐specific aging trajectories. Rather than altering conserved core pathways, such innovations appear to fine‐tune stress response, genome maintenance, and inflammatory programs in a lineage‐dependent manner (Firsanov et al. 2025; Huang et al. 2020; Jiang and Kong 2020; Seluanov et al. 2018). Our findings extend this framework by demonstrating that primate‐specific lncRNAs can interface directly with conserved senescence effectors, thereby providing a mechanistic link between evolutionary novelty and canonical aging pathways. Within this perspective, *LINC01021* exemplifies recently emerged non‐coding regulators that recalibrate the threshold and dynamics of P53 activation to generate species‐restricted aging phenotypes.

*LINC01021* acts as an aging accelerator by lowering the threshold for senescence induction. In the humanized KI mice, constitutive expression of *LINC01021* from the Rosa26 locus under the CAG promoter resulted in increased frailty, depigmentation, and motor coordination deficits, indicating that this primate‐specific lncRNA modulates aging‐related processes in vivo. Immune remodeling emerged more clearly in aged cohorts, and differences in older mice were often trends rather than robust. This pattern supports a model where *LINC01021* advances the onset of functional decline by sensitizing tissues to senescence signals (Di Micco et al. 2021; Sun et al. 2022). Behavioral impairments and immune changes resembling human immunosenescence indicate preferential impact on specific systems (Huang et al. 2021; Sherazi et al. 2023; Zayoud et al. 2024). Partial penetrance and age‐dependent expressivity reflect cross‐species differences (Mattick et al. 2023). Notably, these phenotypes were relatively modest compared with the pronounced senescence responses observed in cultured human fibroblasts. This difference may reflect the gradual accumulation of senescent cells during aging, tissue‐ and organism‐level compensatory mechanisms, and the challenges of modeling a primate‐specific lncRNA within a murine genetic background (Karin and Alon 2021; Ogrodnik 2021; Pal and Tyler 2016). In particular, transgene insertion at the Rosa26 locus may not fully recapitulate the native regulatory environment of the human *LINC01021* locus (Chen et al. 2011), while divergence in downstream regulatory networks could further attenuate its biological effects (Ghanam et al. 2022; Mattick et al. 2023). Consistent with this interpretation, phenotypic alterations were most evident in young adults and became less pronounced in aged cohorts, suggesting that *LINC01021* may accelerate the onset of functional decline rather than drive severe aging phenotypes. Mechanistically, our cellular studies support a model whereby *LINC01021* promotes senescence through the DAZAP1‐RBMX‐P53 pathway. Although direct validation of this mechanism in vivo remains an important direction for future investigation, both DAZAP1 and RBMX have established roles in regulating cell proliferation, development, and tissue homeostasis. DAZAP1‐deficient mice display growth retardation, reproductive defects, and impaired tissue development, indicating an essential role in cellular growth and survival (Hsu et al. 2008). Similarly, RBMX has been implicated in RNA processing, genome stability, and proliferative control, and its disruption leads to defects in development and tissue maintenance (Qiu, Pu, et al. 2025). These observations are consistent with the cellular functions affected by *LINC01021* and suggest that components of the DAZAP1‐RBMX pathway likely contribute to the aging‐like phenotypes observed in *LINC01021* KI mice.

Despite these advances, several considerations highlight the boundaries and future directions of this work. First, the constitutive expression of a human lncRNA in a species lacking endogenous orthologs may amplify certain phenotypes, underscoring the need for tissue‐specific and inducible models to disentangle developmental effects from aging‐associated processes. Second, although our data support a DAZAP1‐RBMX‐P53 regulatory axis, the precise molecular interfaces governing these interactions remain to be defined. Whether *LINC01021* directly scaffolds RNA‐binding proteins or instead reshapes post‐transcriptional regulatory complexes through indirect mechanisms will require detailed structural and biochemical interrogation. Moreover, while *LINC01021* was selected as a representative exemplar, our evolutionary screening uncovered multiple primate‐specific lncRNAs with robust age‐dependent expression patterns. Determining whether these candidates converge on shared senescence pathways or engage distinct regulatory circuits will be essential for delineating the broader architecture of lineage‐restricted aging control.

Altogether, this study proposes that primate‐specific lncRNAs constitute an evolutionarily encoded regulatory layer that interfaces with conserved senescence machinery and may contribute to species‐restricted aging trajectories. By demonstrating that *LINC01021* modulates RBMX stability to influence P53 signaling, thereby promoting cellular senescence and aging‐like phenotypes, our work provides causal evidence that non‐coding regulatory novelty can actively reprogram core aging pathways. More broadly, these findings caution against exclusive reliance on rodent models for dissecting aging mechanisms and highlight the necessity of incorporating lineage‐specific regulators into aging research frameworks. Elucidating such primate‐specific regulatory axes may ultimately enable more precise strategies for modulating senescence and age‐related pathologies within the molecular context of human evolution.

## Author Contributions

Conceptualization: Y.H. Data curation: Y.Z., L.H., and X.D. Formal analysis: Y.Z., X.D., M.Z., C.L., M.G., G.L., and S.P. Funding acquisition: X.D., R.B., G.L., and Y.H. Methodology: Y.Z., L.H., and X.D. Project administration: Y.H., and G.L. Supervision: Y.H. Validation: Y.Z. and L.H. Visualization: Y.Z. and X.D. Writing – original draft: Y.Z. and X.D. Writing – review and editing: A.N., S.K., and Y.H. Investigation: Y.Z., L.H., X.D., and Q.Z.

## Funding

This work was supported by the National Key R&D Program of China (2023YFC3603300 to YHH), National Natural Science Foundation of China (82471599 to YHH, 32500660 to RXB), Yunnan Fundamental Research Projects (202602AS100006 to YHH, 202305AH340006 to YHH, 202401CF070064 to XD), CAS “Light of West China” Program (xbzg‐zdsys‐202312 to YHH), The Postdoctoral Fellowship Program of China Postdoctoral Science Foundation (CPSF) (GZC20232763 to XD), State Key Laboratory Conservation and Utilization of Bio‐Resources in Yunnan (2023KF008 to XD), China Postdoctoral Science Foundation (2025M772550 to RXB), and Yunnan Revitalization Talent Support Program Young Talent Project (GHL). YHH is supported by the Pioneer Hundred Talents Program of the Chinese Academy of Sciences. In addition, YHH is supported by the Yunnan Revitalization Talent Support Program Young Talent Project.

## Ethics Statement

All animal procedures were reviewed and approved by the Animal Ethics Committee of the Kunming Institute of Zoology, Chinese Academy of Sciences (IACUC‐RE‐2025‐08‐008), and were carried out in compliance with institutional guidelines for laboratory animal care and use.

## Consent

The authors have nothing to report.

## Conflicts of Interest

The authors declare no conflicts of interest.

## Supporting information


**Figure S1:** Aging‐associated primate‐conserved lncRNAs across human tissues.
**Figure S2:** *LINC01021* modulates cellular senescence in human fibroblasts.
**Figure S3:** Expression and functional assessment of candidate *LINC01021*‐interacting proteins in senescent cells.
**Figure S4:** Quantitative analyses supporting DAZAP1‐dependent regulation of RBMX by *LINC01021*.
**Figure S5:** Additional phenotypic analyses of *LINC01021*‐humanized transgenic mice.
**Table S1:** List of siRNA sequences used in this study.
**Table S2:** List of primer sequences used in this study.
**Table S3:** List of antibodies used in this study.
**Table S4:** Mouse frailty index scoring criteria.

## Data Availability

The data that support the findings of this study are openly available in ProteomeXchange via PRIDE at https://www.ebi.ac.uk/pride/archive/projects/PXD075354, reference number PXD075354.
