# Supplementary figures and images for: A Primate‐Specific lncRNA LINC01021 Contributes to Cellular and Organismal Aging via DAZAP1‐Dependent Destabilization of RBMX
Source: Aging Cell. 2026 Jun 25;25(7):e70603. doi: 10.1111/acel.70603 (PMC13297028; doi:10.1111/acel.70603)

A

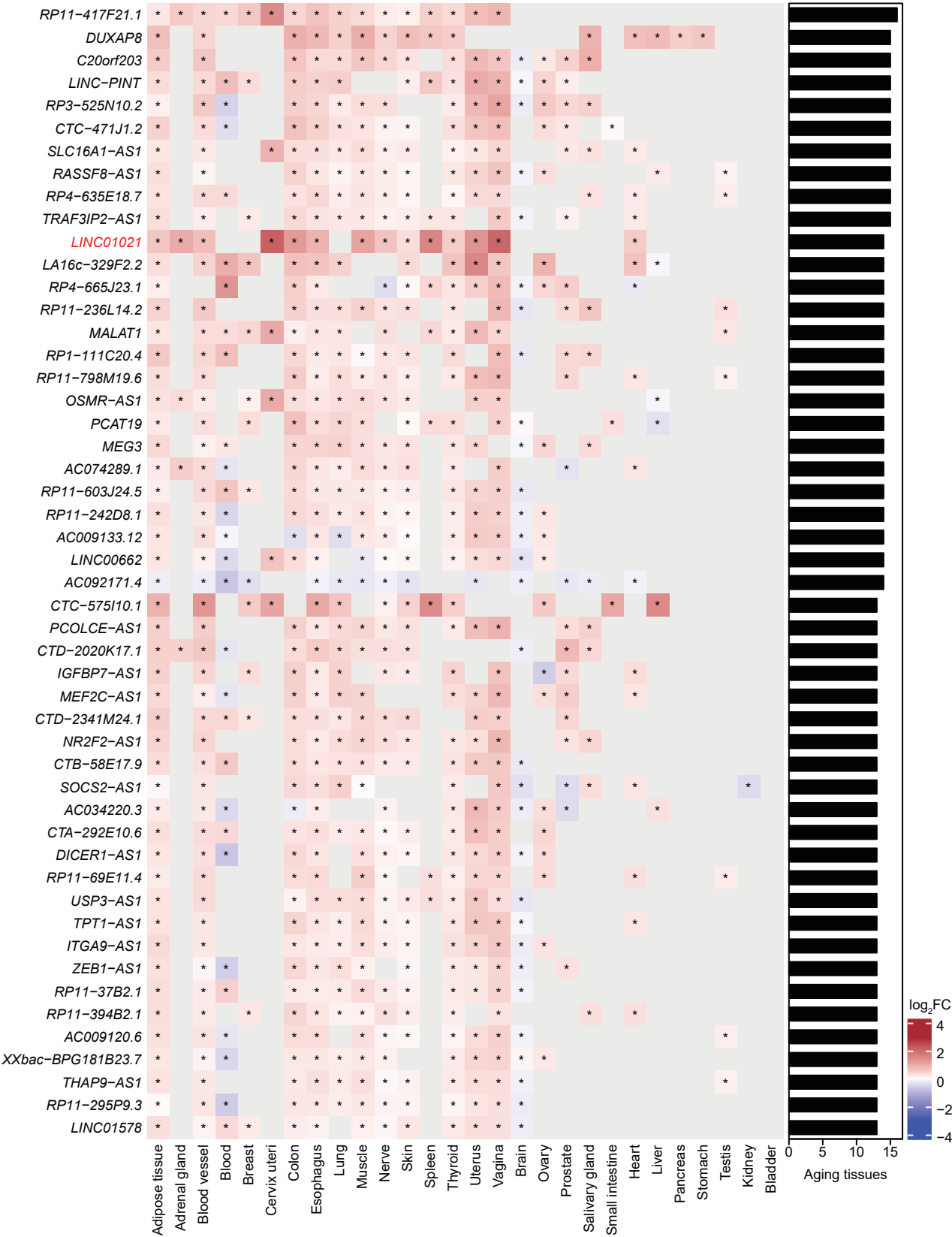

Supplement: Supplementary file 1 — Figure S1: Aging‐associated primate‐conserved lncRNAs across human tissues. Figure S2: LINC01021 modulates cellular senescence in human fibroblasts. Figure S3: Expression and functional assessment of candidate LINC01021‐interacting proteins in senescent cells. Figure S4: Quantitative analyses supporting DAZAP1‐dependent regulation of RBMX by LINC01021. Figure S5: Additional phenotypic analyses of LINC01021‐humanized transgenic mice. Table S1: List of siRNA sequences used in this study. Table S2: List of primer sequences used in this study. Table S3: List of antibodies used in this study. Table S4: Mouse frailty index scoring criteria. [file ACEL-25-e70603-s001.zip › acel70603-sup-0003-FigureS1@Supplementary figure1.pdf]

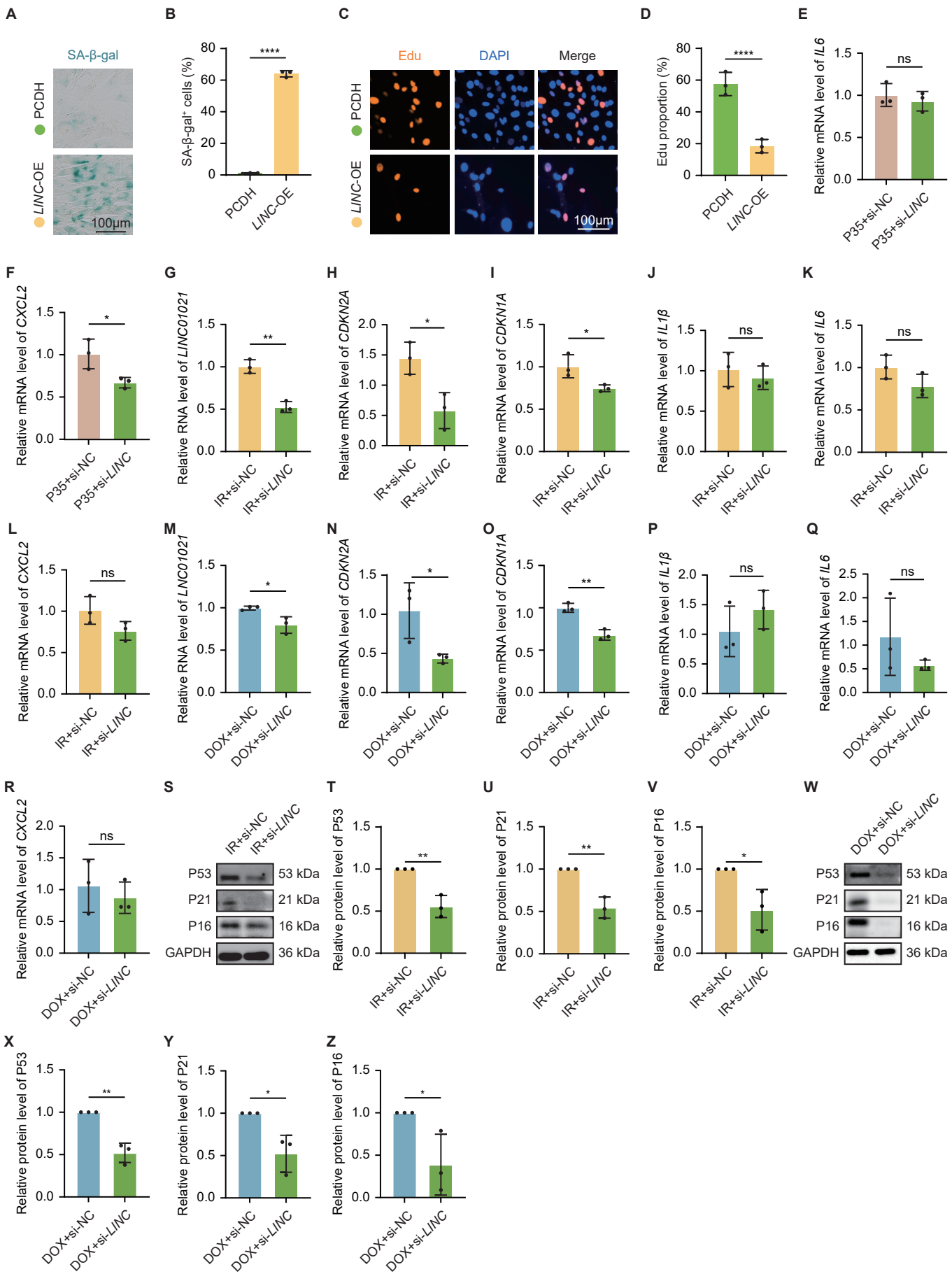

Supplement: Supplementary file 1 — Figure S1: Aging‐associated primate‐conserved lncRNAs across human tissues. Figure S2: LINC01021 modulates cellular senescence in human fibroblasts. Figure S3: Expression and functional assessment of candidate LINC01021‐interacting proteins in senescent cells. Figure S4: Quantitative analyses supporting DAZAP1‐dependent regulation of RBMX by LINC01021. Figure S5: Additional phenotypic analyses of LINC01021‐humanized transgenic mice. Table S1: List of siRNA sequences used in this study. Table S2: List of primer sequences used in this study. Table S3: List of antibodies used in this study. Table S4: Mouse frailty index scoring criteria. [file ACEL-25-e70603-s001.zip › acel70603-sup-0004-FigureS2@Supplementary figure 2.pdf]

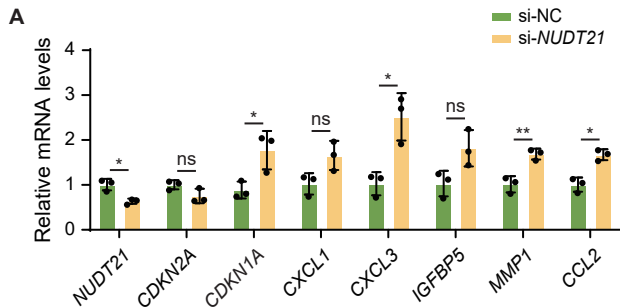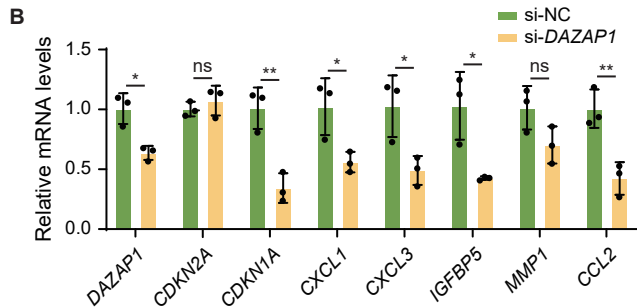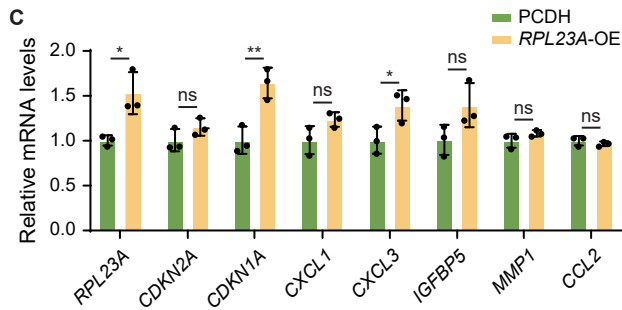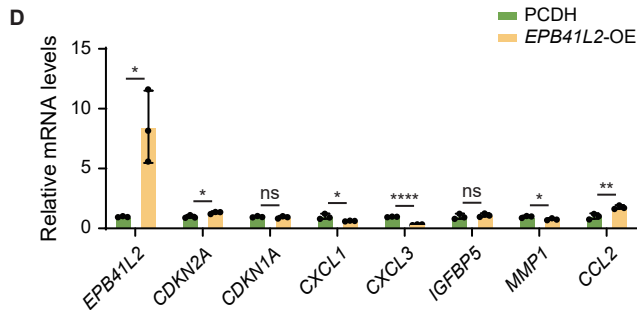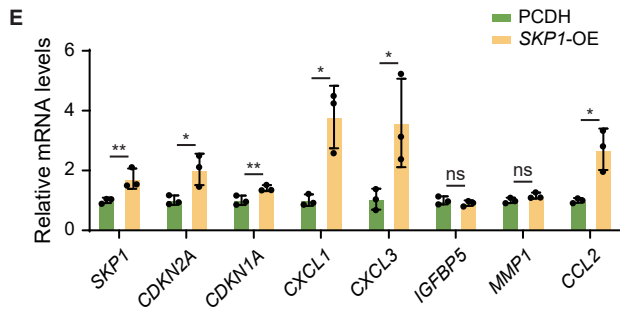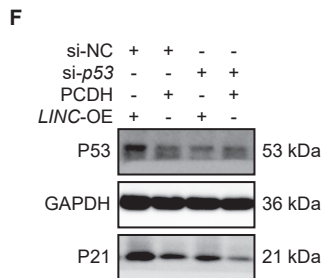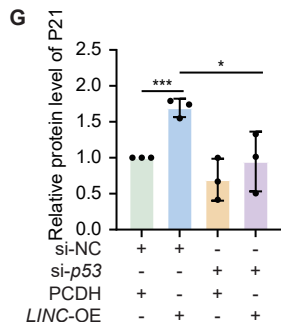

Supplement: Supplementary file 1 — Figure S1: Aging‐associated primate‐conserved lncRNAs across human tissues. Figure S2: LINC01021 modulates cellular senescence in human fibroblasts. Figure S3: Expression and functional assessment of candidate LINC01021‐interacting proteins in senescent cells. Figure S4: Quantitative analyses supporting DAZAP1‐dependent regulation of RBMX by LINC01021. Figure S5: Additional phenotypic analyses of LINC01021‐humanized transgenic mice. Table S1: List of siRNA sequences used in this study. Table S2: List of primer sequences used in this study. Table S3: List of antibodies used in this study. Table S4: Mouse frailty index scoring criteria. [file ACEL-25-e70603-s001.zip › acel70603-sup-0005-FigureS3@Supplementary figure3.pdf]

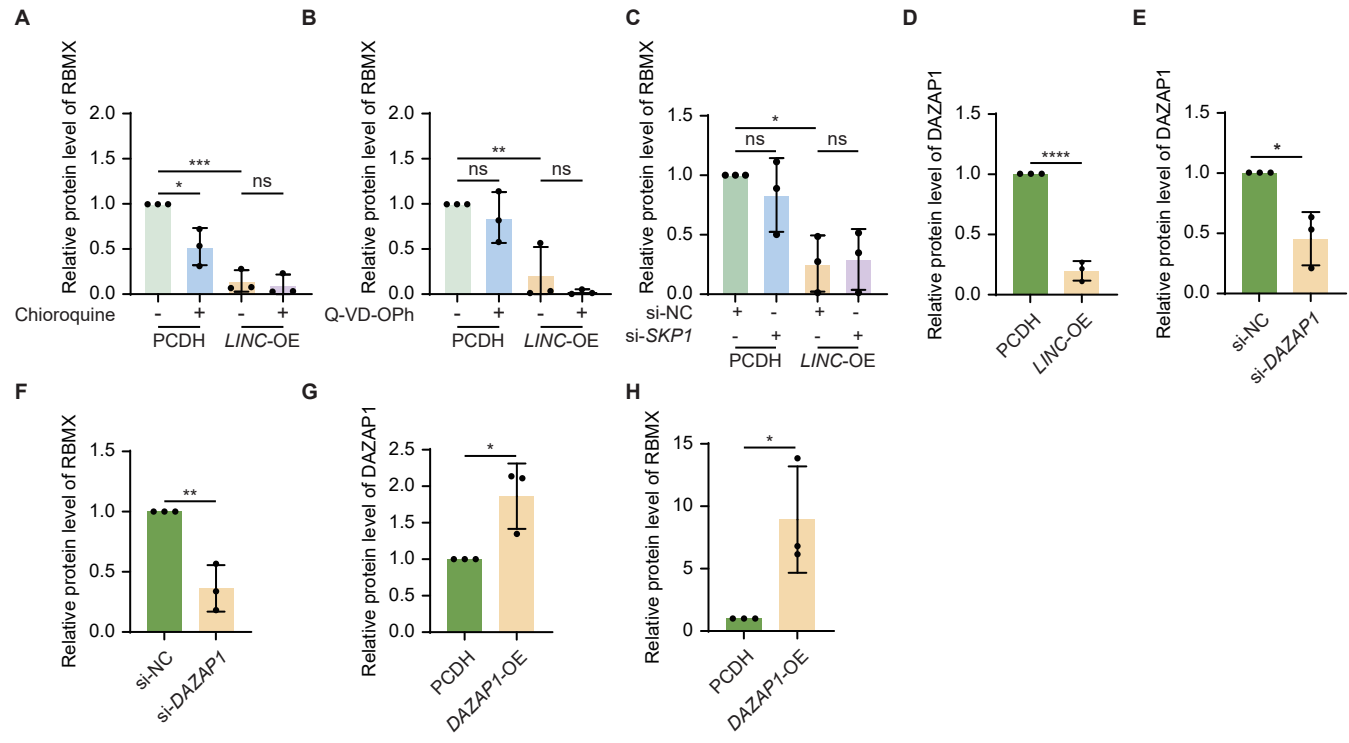

Supplement: Supplementary file 1 — Figure S1: Aging‐associated primate‐conserved lncRNAs across human tissues. Figure S2: LINC01021 modulates cellular senescence in human fibroblasts. Figure S3: Expression and functional assessment of candidate LINC01021‐interacting proteins in senescent cells. Figure S4: Quantitative analyses supporting DAZAP1‐dependent regulation of RBMX by LINC01021. Figure S5: Additional phenotypic analyses of LINC01021‐humanized transgenic mice. Table S1: List of siRNA sequences used in this study. Table S2: List of primer sequences used in this study. Table S3: List of antibodies used in this study. Table S4: Mouse frailty index scoring criteria. [file ACEL-25-e70603-s001.zip › acel70603-sup-0006-FigureS4@Supplementary figure 4.pdf]

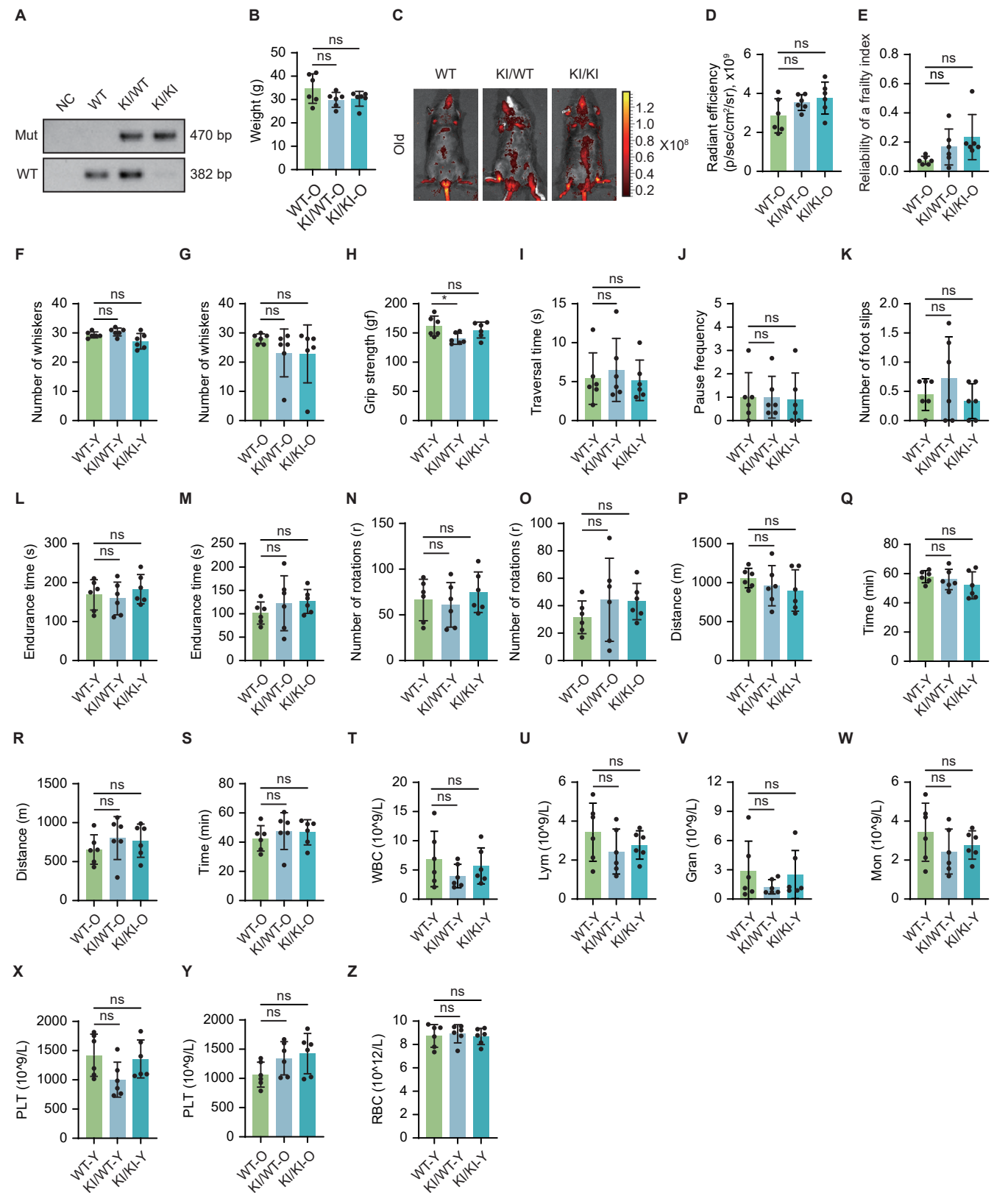

Supplement: Supplementary file 1 — Figure S1: Aging‐associated primate‐conserved lncRNAs across human tissues. Figure S2: LINC01021 modulates cellular senescence in human fibroblasts. Figure S3: Expression and functional assessment of candidate LINC01021‐interacting proteins in senescent cells. Figure S4: Quantitative analyses supporting DAZAP1‐dependent regulation of RBMX by LINC01021. Figure S5: Additional phenotypic analyses of LINC01021‐humanized transgenic mice. Table S1: List of siRNA sequences used in this study. Table S2: List of primer sequences used in this study. Table S3: List of antibodies used in this study. Table S4: Mouse frailty index scoring criteria. [file ACEL-25-e70603-s001.zip › acel70603-sup-0007-FigureS5@Supplementary figure5.pdf]
