# Supplementary material for: A Primate‐Specific lncRNA LINC01021 Contributes to Cellular and Organismal Aging via DAZAP1‐Dependent Destabilization of RBMX
Source: Aging Cell. 2026 Jun 25;25(7):e70603. doi: 10.1111/acel.70603 (PMC13297028; doi:10.1111/acel.70603)
Supplement: Supplementary file 1 — Figure S1: Aging‐associated primate‐conserved lncRNAs across human tissues. Figure S2: LINC01021 modulates cellular senescence in human fibroblasts. Figure S3: Expression and functional assessment of candidate LINC01021‐interacting proteins in senescent cells. Figure S4: Quantitative analyses supporting DAZAP1‐dependent regulation of RBMX by LINC01021. Figure S5: Additional phenotypic analyses of LINC01021‐humanized transgenic mice. Table S1: List of siRNA sequences used in this study. Table S2: List of primer sequences used in this study. Table S3: List of antibodies used in this study. Table S4: Mouse frailty index scoring criteria. [file ACEL-25-e70603-s001.zip › acel70603-sup-0001-FigureS1-S5-TableS1-S4.doc]

**A primate-specific lncRNA *LINC01021* contributes to cellular and organismal aging via DAZAP1-dependent destabilization of RBMX**

Yan Zhang^1,2,#^, Li Hu^1,2,#^, Xin Dong^1,#^, Qinghua Zeng^1^, Meiting Zi^1^, Ayesha Nisar^1,2^, Sawar Khan^3,4^, Raoxian Bai^1^, Chonghui Liu^5^, Mingxia Ge^6^, Shaoyan Pu^7^, Gonghua Li^1,*^, Yonghan He^1,2,8*^

^1^State Key Laboratory of Genetic Evolution & Animal Models, Key Laboratory of Healthy Aging Research of Yunnan Province, Kunming Institute of Zoology, Chinese Academy of Sciences, Kunming 650201, China

^2^University of Chinese Academy of Sciences, Beijing 100101, China

^3^Department of Cell Biology, School of Life Sciences, Central South University, Changsha 410013, China

^4^Institute of Molecular Biology and Biotechnology, The University of Lahore, Lahore 54000, Pakistan

^5^State Key Laboratory of Primate Biomedical Research, Institute of Primate Translational Medicine, Kunming University of Science and Technology, Kunming 650201, China

^6^School of Basic Medical Sciences, Wannan Medical College, Wuhu 241002, China

^7^Biodiversity Data Center of Kunming Institute of Zoology, Chinese Academy of Sciences, Kunming 650201, China

^8^KIZ/CUHK Joint Laboratory of Bioresources and Molecular Research in Common Diseases, Kunming 650201, China

^#^These authors contributed equally to the work.

*Corresponding author:

Yonghan He, M.D., Professor, Principal Investigator

State Key Laboratory of Genetic Evolution and Animal Models, Kunming Institute of Zoology, Chinese Academy of Sciences

Phone: +86-871-65118976

E-mail: heyonghan@mail.kiz.ac.cn

Or

Gonghua Li, Professor

State Key Laboratory of Genetic Evolution and Animal Models, Kunming Institute of Zoology, Chinese Academy of Sciences

Phone: +86-871-68125401

E-mail: ligonghua@mail.kiz.ac.cn

**Supplementary Figures**

**Supplementary Figure 1.** Aging-associated primate-conserved lncRNAs across human tissues.

**Supplementary Figure 2.** *LINC01021* modulates cellular senescence in human fibroblasts.

**Supplementary Figure 3.** Expression and functional assessment of candidate *LINC01021*-interacting proteins in senescent cells.

**Supplementary Figure 4.** Quantitative analyses supporting DAZAP1-dependent regulation of RBMX by *LINC01021*.

**Supplementary Figure 5.** Additional phenotypic analyses of *LINC01021*-humanized transgenic mice.

**Supplementary Tables**

**Supplementary Table 1.** List of siRNA sequences used in this study

**Supplementary Table 2.** List of primer sequences used in this study

**Supplementary Table** **3.** List of antibodies used in this study

**Supplementary Table 4.** Mouse frailty index scoring criteria

**Figure S1.** Aging-associated primate-conserved lncRNAs across human tissues. **(A)** Heatmap showing log₂FC (aged vs. young) of primate-conserved, aging-associated lncRNAs across human tissues. An adjacent bar plot summarizes the number of tissues in which each lncRNA exhibits significant age-associated expression changes (adjusted *p* < 0.05), highlighting the prevalence of lncRNAs with multi-tissue aging associations.

**Figure S2.** *LINC01021* modulates cellular senescence in human fibroblasts. **(A, B)** Representative SA-β-gal staining and quantification of SA-β-gal positive cells in *LINC01021*-OE HDF cells. **(C, D)** Representative EdU staining and quantification of EdU-positive cells in *LINC01021*-OE HDF cells. **(E, F)** qRT-PCR analysis of SASP-associated genes in replicative senescent HELF cells following *LINC01021* knockdown. **(G-L)** qRT-PCR of *LINC01021*(G), *CDKN2A* (H), *CDKN1A* (I), and SASP-associated genes (J-L) in IR-induced senescent HELF cells following *LINC01021* knockdown. **(M-R)** qRT-PCR analysis of *LINC01021*(M), *CDKN2A* (N), *CDKN1A* (O), and SASP-associated genes (P-R) in DOX-induced senescent HELF cells following *LINC01021* knockdown. **(S-V)** Western blot analysis and quantification of P53, P21, and P16 protein levels in IR-induced senescent HELF cells after *LINC01021* knockdown. **(W-Z)** Western blot analysis and quantification of P53, P16, and P21 protein levels in DOX-induced senescent HELF cells after *LINC01021* knockdown. Data are shown as mean ± SEM from at least three independent experiments.

**Figure S3.** Expression and functional assessment of candidate *LINC01021*-interacting proteins in senescent cells. **(A)** qRT-PCR analysis of *NUDT21*, *CDKN2A*, *CDKN1A*, and SASP genes in si-*NUDT21* HELF cells. **(B)** qRT-PCR analysis of *DAZAP1*, *CDKN2A*, *CDKN1A*, and SASP genes in si-*DAZAP1* HELF cells. **(C)** qRT-PCR analysis of *RPL23A*, *CDKN2A*, *CDKN1A*, and SASP genes in *RPL23A*-OE HELF cells. **(D)** qRT-PCR analysis of *EPB41L2*, *CDKN2A*, *CDKN1A*, and SASP genes in *EPB41L2*-OE HELF cells. **(E)** qRT-PCR analysis of *SKP1*, *CDKN2A*, *CDKN1A*, and SASP genes in *SKP1*-OE HELF cells. **(F)** *p53* depletion attenuates *LINC01021*-induced activation of the P53/P21 signaling pathway. **(G)** Quantification of P21 protein levels shown in Fig. S3F. Data are shown as mean ± SEM from at least three independent experiments.

**Figure S4.** Quantitative analyses supporting DAZAP1-dependent regulation of RBMX by *LINC01021*. **(A-C)** Quantification of RBMX protein levels shown in Fig. 5H-J, respectively. **(D)** Quantification of DAZAP1 protein levels shown in Fig. 5L. **(E, F)** Quantification of DAZAP1 and RBMX protein levels shown in Fig. 5M. **(G, H)** Quantification of DAZAP1 and RBMX protein levels shown in Fig. 5P. Data are shown as mean ± SEM from at least three independent experiments.

**Figure S5.** Additional phenotypic analyses of *LINC01021*-humanized transgenic mice. **(A)** PCR-based genotyping of WT and KI mice. **(B)** Body weight of aged mice. **(C, D)** Representative NIR fluorescence imaging using a SA-β-gal responsive probe and quantification of fluorescence intensity in aged mice. **(E)** Frailty index in aged mice. **(F, G)** Whisker number in young (F) and aged (G) mice. **(H)** Forelimb grip strength in young mice. **(I-K)** Balance and coordination performance assessed by balance beam traversal time (I), pause frequency (J), and foot slips (K) in young mice. **(L-O)** Rotarod performance in young (L, N) and aged (M, O) mice, including endurance time (L, M) and rotation number (N, O). **(P-S)** Treadmill fatigue test performance in young (P, Q) and aged (R, S) mice, including running distance (P, R) and running time (Q, S). **(T-X)** Hematological parameters in young mice, including white blood cells (WBC), lymphocytes, granulocytes, monocytes, and platelets. **(Y, Z)** Platelet counts in aged mice (Y) and red blood cell (RBC) counts in young mice (Z).

**Table S1. List of siRNA sequences used in this study**

| siRNA | siRNA sequences (5’-3’) | Company |
| --- | --- | --- |
| si-*PURPL* | CCAGCATCCTCTAATTATT | RiboBio |
| si-*p53* | GAGGUUGGCUCUGACUGUACCACCA | Sangon Biotech |
| si-*RBMX*-sense | CGAAGUGAUCUCUACUCAA/dT//dT/ | Sangon Biotech |
| si-*RBMX*-antisense | UUGAGUAGAGAUCACUUCG/dT//dT/ | Sangon Biotech |
| si-*NUDT21*-sense | AGUACAUCCAGCAGACGAA/dT//dT/ | Sangon Biotech |
| si-*NUDT21*-antisense | UUCGUCUGCUGGAUGUACU/dT//dT/ | Sangon Biotech |
| si-*DAZAP1*-sense | CGGAGGUAGUCAUGAUCUA/dT//dT/ | Sangon Biotech |
| si-*DAZAP1*-antisense | UAGAUCAUGACUACCUCCG/dT//dT/ | Sangon Biotech |
| si-NC-sense | UUCUCCGAACGUGUCACGUTT | Sangon Biotech |
| si-NC-antisense | ACGUGACACGUUCGGAGAATT | Sangon Biotech |

| **Table S2.** List of primer sequences used in this study | | |
| --- | --- | --- |
| Primers | Primer sequences (5’-3’) | Company |
| *GAPDPH*-F | ATTCCATGGCACCGTCAAGG | Sangon Biotech |
| *GAPDPH*-R | TCGCCCCACTTGATTTTGGA | Sangon Biotech |
| *CDKN2A*-F | GGGAGGGCTTCCTGGACA | Sangon Biotech |
| *CDKN2A*-R | CAATCGGGGATGTCTGAGGG | Sangon Biotech |
| *CDKN1A*-F | TGTCCGTCAGAACCCATGC | Sangon Biotech |
| *CDKN1A*-R | AAAGTCGAAGTTCCATCGCTC | Sangon Biotech |
| *LINC01021*-F | TGTGAATTTAGGCCTACGTGA | Sangon Biotech |
| *LINC01021*-R | AGCCCCTCATTCGTTATATTTT | Sangon Biotech |
| *IL1β*-F | AGCTACGAATCTCCGACCAC | Sangon Biotech |
| *IL1β*-R | CGTTATCCCATGTGTCGAAGAA | Sangon Biotech |
| *IL6*-F | ACTCACCTCTTCAGAACGAATTG | Sangon Biotech |
| *IL6*-R | CCATCTTTGGAAGGTTCAGGTTG | Sangon Biotech |
| *CXCL2*-F | GAAAGCTTGTCTCAACCCCG | Sangon Biotech |
| *CXCL2*-R | TGGTCAGTTGGATTTGCCATTTT | Sangon Biotech |
| *CXCL1*-F | CAGGGAATTCACCCCAAGAACA | Sangon Biotech |
| *CXCL1*-R | GGATGCAGGATTGAGGCAAGC | Sangon Biotech |
| *CXCL3*-F | ACCGAAGTCATAGCCACACTC | Sangon Biotech |
| *CXCL3*-R | GGTGCTCCCCTTGTTCAGTAT | Sangon Biotech |
| *IGFBP5*-F | AGATGGCCGAGGAGACCTAC | Sangon Biotech |
| *IGFBP5*-R | GCGGTCCTTCTTCACTGCTT | Sangon Biotech |
| *MMP1*-F | GCTGCTTACGAATTTGCCGA | Sangon Biotech |
| *MMP1*-R | GGGGTATCCGTGTAGCACAT | Sangon Biotech |
| *CCL2*-F | CAGCCAGATGCAATCAATGCC | Sangon Biotech |
| *CCL2*-R | TGGAATCCTGAACCCACTTCT | Sangon Biotech |
| *NUDT21*-F | GGTCACTCAGTTCGGCAACAA | Sangon Biotech |
| *NUDT21*-R | CTCATGCGCTGAAATCTGGC | Sangon Biotech |
| *RPL23A*-F | TGCTATCATCAAATTCCCAC | Sangon Biotech |
| *RPL23A*-R | ACATACGCCTTCTTCTCTCC | Sangon Biotech |
| *EPB41L2*-F | AAACATGCCAAGGGACAAGTG | Sangon Biotech |
| *EPB41L2*-R | TCTAACCAGTTTTTCTGCTCAGG | Sangon Biotech |
| *SKP1*-F | ATGCCTACGATAAAGTTGCAGA | Sangon Biotech |
| *SKP1*-R | TCCATTCCCAAATCTTCCAGC | Sangon Biotech |
| *FBL*-F | TGACATCGTTGGTCCGGATG | Sangon Biotech |
| *FBL*-R | TGGGCCACATCAGCAAAGAT | Sangon Biotech |
| *DAZAP1*-F | GCGGTCTTGACTGGAGCA | Sangon Biotech |
| *DAZAP1*-R | TTCGGCCATCTAGCGTGT | Sangon Biotech |
| *RBMX*-F | TGGAAGCAGTCGCTATGATG | Sangon Biotech |
| *RBMX*-R | GAGGGTACCCCCTTTCCATA | Sangon Biotech |
| *DKC1*-F | ATGGCGGATGCGGAAGTAAT | Sangon Biotech |
| *DKC1*-R | CCACTGAGACGTGTCCAACT | Sangon Biotech |
| *LINC01021*-promotor-F | GTAGAGACGGGGAACTTTGGA | Sangon Biotech |
| *LINC01021*-promotor-R | GCTTTTCCACTCTGTGAATGGG | Sangon Biotech |
| WT-F | CCAAATTCTTCACAGCCAAAG | Sangon Biotech |
| WT-R | TTGTGTGCTCAGCAAGTC | Sangon Biotech |
| Mut-F | CACATTGAAGGGTCAGTAGAG | Sangon Biotech |
| Mut-R | AGGTAAGAACCTGGGATACA | Sangon Biotech |

**Table S3. List of antibodies used in this study**

| Antibodies | Company | Catalog# | Concentration |
| --- | --- | --- | --- |
| P16 | Santa cruz Biotechnology | sc-1661 | 1:1000 |
| P21 | Cell Signaling Technology | 37543S | 1:1000 |
| P53 | Cell Signaling Technology | 9282T | 1:1000 |
| RBMX | Selleckchem | F1965 | 1:1000 |
| DAZAP1 | UpingBio | YP-mAb-18681 | 1:1000 |
| GAPDH | Beyotime Biotechnology | AF1186 | 1:1000 |
| Anti-Rabbit IgG (H+L) HRP | Beyotime Biotechnology | A0208 | 1:2000 |
| Anti-Mouse IgG (H+L) HRP | Beyotime Biotechnology | A0216 | 1:2000 |

**Table S4.** Mouse frailty index scoring criteria

| Item | Scoring Criteria |
| --- | --- |
| Alopecia | 0: Normal coat density;0.5: ≤ 25% hair loss;1: ≥ 25% hair loss |
| Coat Color Loss | 0: Normal; 0.5: Patchy discoloration 1: ≥ 25% grey/white area |
| Dermatitis | 0: None; 0.5: Localized; 1: Extensive |
| Coat Condition | 0: Glossy; 0.5: Slightly rough; 1: Ruffled |
| Piloerection | 0: None; 0.5: Neck only; 1: Widespread |
| Cataracts | 0: None; 0.5: Mild opacity; 1: Pronounced opacity |
| Ocular Discharge/Swelling | 0: None; 0.5: Mild; 1: Severe |
| Microphthalmia | 0: Normal; 0.5: Slightly small; 1: Pronounced |
| Corneal Opacity | 0: None; 0.5: Minimal; 1: Obvious opacity |
| Tumors | 0: None; 0.5: ≤ 1.0 cm; 1: ≥ 1.0 cm |
| Abdominal Distension | 0: None; 0.5: Mild; 1: Distinct ‘W’ shape |
| Kyphosis | 0: None; 0.5: Moderate; 1: Severe |
| Malocclusion | 0: Normal; 0.5: Mild uneven; 1: Severe |
| Rectal Prolapse | 0: None; 0.5: Mild; 1: Severe |
| Genital Prolapse | 0: None; 0.5: Mild; 1: Obvious |
| Whisker Loss | 0-1: SD-based scoring |
| Gait Disorder | 0: Normal; 0.5: Abnormal gait; 1: Impaired mobility |
| Tremor | 0: None; 0.5: Mild; 1: Severe |
| Hearing Loss | 0: Always responsive; 0.5: Partial response; 1: No response |
| Vision Loss | 0: > 4 cm; 0.5: 2-4 cm; 1: < 2 cm |
| Threat Reflex | 0: Full response; 0.5: Partial; 1: None |
| Vestibular Dysfunction | 0: None; 0.5: Mild ;1: Severe |
| Tail Stiffness | 0: None; 0.5: Mild stiffness; 1: Rigid |
